# Morbidity and medication use preceding a diagnosis of late-onset Alzheimer’s disease: a Danish nationwide study

**DOI:** 10.1007/s00415-026-13967-y

**Published:** 2026-07-02

**Authors:** Cecilia El-Sayed Petersen, Line Damsgaard, Janet Janbek, Thomas Munk Laursen, Karsten Vestergaard, Hanne Gottrup, Gunhild Waldemar

**Affiliations:** 1 Danish Dementia Research Centre, Section 8008, Department of Neurology, Copenhagen University Hospital, Rigshospitalet, Blegdamsvej 9, 2100 Copenhagen, Denmark; 2 Department of Economics and Business Economics, National Centre for Register-Based Research, Aarhus BSS, Aarhus University, Fuglesangs Allé 26- Building R, 8210, Aarhus, Denmark; 3 Department of Neurology, Dementia Clinic, Aalborg University Hospital, Ladegaardsgade 5, 9000 Aalborg, Denmark; 4 Department of Neurology, Dementia Clinic, Aarhus University Hospital, Palle Juul-JensenBoulevard 99, 8200 Aarhus, Denmark; 5 Department of Clinical Medicine, University of Copenhagen, Blegdamsvej 3B, 2200 Copenhagen, Denmark

**Keywords:** Alzheimer’s disease, Late-onset Alzheimer’s disease, Prodromal features, Morbidity, Medication use, Temporality

## Abstract

**Introduction:**

Symptoms of Alzheimer’s disease can emerge years before diagnosis, but detailed temporal mapping of morbidity and medication use in the prodromal period is lacking.

**Methods:**

We conducted a nationwide retrospective, incidence density-matched nested case–control study including individuals diagnosed with late-onset Alzheimer’s disease in Danish memory clinics from 2016 to 2022 and cognitively healthy matched controls. Exposures were primary and secondary discharge diagnoses from hospital contacts, and redeemed prescriptions.

**Results:**

A total of 19,102 patients and 57,306 matched controls were included. At 10 years before diagnosis, individuals who later developed late-onset Alzheimer’s disease had more mental health and behavioral diagnoses and increased use of psychopharmaceuticals, as well as more injuries and hospital contacts for non-specific symptoms. During the five years and one-year preceding diagnosis, morbidity increased across multiple diagnostic categories, while most medication categories decreased.

**Conclusion:**

Our findings suggest that prodromal late-onset Alzheimer’s disease is characterized by gradual accumulation of morbidity, with injuries and neuropsychiatric symptoms as the most persistent. This knowledge can guide future hypothesis-driven studies towards better characterization of the prodromal phase that may improve early detection, reduce the burden of unexplained symptoms experienced by patients and their caregivers, and support clinicians facing diagnostic uncertainty.

**Supplementary Information:**

The online version contains supplementary material available at 10.1007/s00415-026-13967-y.

## Background

Alzheimer’s disease (AD) is a neurodegenerative disease characterized by cognitive impairment and functional decline[1]. As the most common form of dementia, AD contributes to 60–70% of cases [2]. While research shows a decline in age-specific incidence, the global burden of AD continues to rise due to increasing life expectancy and demographic shifts [3, 4].

The underlying neuropathology is characterized by the accumulation of amyloid-beta plaques and tau neurofibrillary tangles [5] developing years, likely even decades, before onset of clinical symptoms [5]. This slow development indicates a long prodromal phase where signs and symptoms are likely to emerge gradually. Studies have shown that cognitive symptoms occur as early as eight [6] to 12 [7] years before the AD diagnosis with depressive symptoms and semantic complaints among the earliest signs [7, 8], but there is still limited understanding about early and potentially non-cognitive symptoms that may appear during this prodromal phase. Such knowledge could aid in efforts towards timely diagnosis, which is crucial, not only for enabling timely intervention and planning but also to reduce the financial, health, and emotional burdens on patients, caregivers, and the healthcare system[9]. Timely diagnosis is particularly relevant given recent advancements in disease-modifying treatments that target early stages of AD [10, 11]. Despite this knowledge, dementia remains undiagnosed in 50–90% of cases across the world [12–14], and when diagnosed, it is often done so at later stages. The 2024 report from the Danish Dementia Quality Database (DanDem) shows that 57% of the patients diagnosed with dementia at Danish memory clinics were diagnosed at moderate-severe stages [15].

Previous observational studies have investigated the association between selected prodromal features, predefined risk factors, and medications prior to diagnosis of all-cause dementia[16–18], and specifically AD [19–22]. These studies identified several diseases and symptoms which were associated with subsequent dementia or AD with the highest prevalence found for psychiatric disorders such as depression and anxiety, implicating these as likely early signs—or early consequences—of dementia. However, existing research on prodromal features of dementia has been limited by small sample sizes, reliance only on primary care data or selected populations, focus on predefined factors, and the lack of assessment of temporal changes.

Therefore, nationwide longitudinal studies that comprehensively assess all diseases/conditions and their temporal emergence are needed to provide evidence on characteristics of the prodromal phase. To address the gap in the existing literature, this nationwide study aims to describe potential patterns characteristic of the prodromal phase of late-onset Alzheimer’s disease (LOAD) by exploring whether individuals diagnosed with LOAD differ from cognitively healthy, matched adults in terms of hospital contacts and prescription medication use in the 10 years preceding diagnosis.

## Methods

### Data sources

Cases were identified from DanDem [23]. DanDem contains information from all secondary healthcare facilities that accept referrals for diagnostic evaluation of cognitive impairment and dementia from 2016 and onward. DanDem records information on etiology, severity of dementia at time of diagnosis, diagnostic investigations performed, and results of cognitive tests. Reasons for hospital contacts were drawn from the Danish National Patient Register (DNPR) [24] and the Danish Psychiatric Central Research Register (DPCRR) [25], which contain information on all in- and outpatient contacts. Information on redeemed prescriptions was drawn from the Danish National Prescription Registry (DNPrR) [26]. The Population Education Register [27] and the Civil Registration System [28] were used for information on covariates. Information on nursing home residence was drawn from the Nursing Home Data Register [29].

### Study design and population

We conducted a retrospective incidence-density matched nested case–control study. The study population included all individuals diagnosed with AD in a Danish memory clinic between 2016 and 2022. Matched controls were drawn from a nationwide cohort of all Danish citizens. We followed the same methodology of our previous published studies on young-onset Alzheimer’s disease [30, 31].

#### Case definition

LOAD is defined as dementia due to AD with symptom onset after age 65 [32]. As information on symptom onset is not available in the registry data, we used age at diagnosis as a proxy. Based on previous literature suggesting an average delay of five years between symptom onset and clinical diagnosis [33–35], we restricted inclusion to individuals ≥ 70 years at time of diagnosis. Cases were identified from DanDem as individuals with a first recorded diagnosis of dementia due to AD between the start of register (2016) and 2022. Diagnoses were established according to the National Institute on Aging-Alzheimer’s Association (NIA-AA) workgroups 2011 criteria [36] and disease severity was classified using the International Classification of Diseases 10th revision (ICD-10) [32]. Index date was defined as the date of AD diagnosis.

#### Control definition

Each case was matched to three controls from the full risk set, defined as the entire Danish population, based on sex and age (date of birth ± 90 days). At time of matching, all controls were at risk of a first diagnosis of AD. Thus, controls were selected using incidence density sampling, allowing the odds ratios estimated from conditional logistic regression to be interpreted as incidence rate ratios (IRR) [37, 38]. Controls were randomly selected on the following criteria: no dementia record, no entry in DanDem prior to index date, and no redeemed prescription for antidementia medication before index date (see supplementary Table S1 for details).

#### Overall exclusion criteria

To ensure completeness of data, cases and controls who did not live in Denmark throughout the study period were excluded. Exclusion criteria and specific diagnostic codes used to determine these can be seen in Table S1.

### Definition of exposures

#### Morbidity

We defined morbidity using primary and secondary discharge diagnoses from in- and outpatient hospital contacts drawn from DNPR and DPCRR. We classified diagnoses according to the main chapters of the ICD-10 system (Chapters I–XIV, XVIII–XIX, and XXI) and their subcategories. These subcategories were based either on predefined ICD-10 subcategories or on broader groupings decided by the research team (Table S2).

We considered individuals exposed if they had received at least one diagnosis within a category or subcategory during the 10 years preceding the index date. We excluded diagnoses related to pregnancy, childbirth or the perinatal period, and congenital malformations and chromosomal abnormalities (ICD-10 chapters XV/0, XVI/P, XVII/Q). Within each category, we also identified the three most prevalent diagnoses on a three-digit level (e.g., A00) in the study population.

#### Medication

We defined medication use based on redeemed prescriptions in the DNPrR. We categorized medications according to overall category corresponding to the Anatomical Therapeutic Chemical (ATC) main groups, and subcategories based on indication. We primarily based these subcategories on ATC therapeutic classifications, but we included more specific three- or four-digit ATC-codes where relevant. Table S3 provides the definitions of overall categories, subcategories, and their corresponding ATC codes.

We considered individuals exposed if they had redeemed at least one prescription during the 10 years preceding the index date. Within each overall category, we also identified the three most frequently redeemed prescription medications (defined at the five-digit level, e.g., A01AA) in the study population.

If fewer than 5% of the study population had a diagnosis or redeemed prescription within a specific subcategory during the 10-year study period, we combined these diagnoses or prescriptions into an “other” category within the corresponding overall category for the regression analysis, as analyses based on very small numbers would not provide meaningful comparisons.

### Covariates

We included covariates on age, sex, living status at index date (living alone, living with someone, or living in a nursing home), and highest attained educational level at age 50 years. Educational level was categorized in accordance with the International Standard Classification of Education (ISCED) [39] as low (primary, and lower secondary), medium (upper secondary, and short cycle tertiary), and high (Bachelor, Master, and Doctoral or equivalent).

### Data analysis

#### Main analysis

For each morbidity and medication category, we used conditional logistic regression models to investigate the association between a diagnosis of LOAD and having at least one diagnosis/one redeemed prescription within each overall category, subcategory, and the three most frequent diagnoses/medications within each overall category (Table S2, S3). Each matched set served as a stratum in the regression models. IRRs were calculated for three time intervals preceding the index date: a) 10- > 5 years, b) 5- > 1 year, and c) ≤ 1 year before index date. For subcategories, overall IRRs were calculated without stratification in time intervals.

As we have previously found psychiatric morbidity and medication use to be significantly increased prior to a diagnosis of young-onset Alzheimer’s disease [30, 31], we decided a priori to examine the morbidity subcategory Mental and behavioral disorders category and medication subcategory Nervous system in the three time-intervals previously described.

We conducted two adjustment models: one unadjusted, and one adjusted for age, sex, highest attained educational level at age 50 years, and living status at index date. All results are presented with 95% confidence intervals (CI), corresponding to a 5% significance level. As this study is exploratory, the findings should not be interpreted as causal. Given the large number of associations examined, some findings may be due to chance. The results should therefore be viewed as a basis for generating hypotheses for future research. Further studies are needed to assess whether the observed associations reflect causal relationships, using appropriate methodological approaches.

#### Sensitivity analysis

In a sensitivity analysis, we stratified by 1) disease severity at index date (mild dementia, moderate/severe dementia), 2) sex, and 3) age at index date (70–80 years, > 80 years). Furthermore, to account for potential delay in registration of LOAD diagnosis in DanDem, we conducted a sensitivity analysis censoring all diagnoses and prescriptions within six months of diagnosis, i.e., limiting the ≤ 1-year interval to ≤ 1-year to > 6 months prior to diagnosis. All sensitivity analyses were conducted assessing morbidity and medication use in overall categories and only adjusted IRRs are presented.

Data analyses were performed using SAS 9.4 software. This research project was approved by the Danish Data Protection Agency, Statistics Denmark, and the Danish Health Data Authority. Danish law does not require ethics committee approval or informed patient consent.

## Results

### Baseline characteristics

A total of 19,102 patients diagnosed with LOAD in DanDem between 2016 and 2022 were included in the study, along with 57,306 sex and age-matched controls. Covariates were evenly distributed among cases and controls. At the time of diagnosis, 47% of the cases had mild dementia, 45% had moderate, and 8% had severe dementia. Among the cases and controls, 63% were female, 50% were between 80 and 90 years old, and the majority were living alone or with someone. Residence in nursing homes was more common among cases (7%) than controls (3%) (Table 1).

**Table 1:** Baseline characteristics of study population

	Cases (*n*: 19,102)	Controls (*n*: 57,306)
Age at index date		
70–80 years	7916 (41%)	23,739 (41%)
80–90 years	9568 (50%)	28,713 (50%)
> 90 years	1618 (8%)	4854 (8%)
Sex, *n* (%)		
Male	7058 (37%)	21,174 (37%)
Female	12,044 (63%)	36,132 (63%)
Dementia syndrome severity at time of diagnosis, *n* (%)		
Mild dementia	8941 (47%)	
Moderate dementia	8628 (45%)	
Severe dementia	1533 (8%)	
Cognitive examination scores at first visit^a^, median		
MMSE	21	
ACE	62	
Educational attainment at age 50, n (%)		
Low	8040 (42%)	25,020 (44%)
Medium	7389 (39%)	21,376 (37%)
High	3143 (16%)	9240 (16%)
Unknown	530 (3%)	1670 (3%)
Living status at time of diagnosis, *n* (%)		
Living alone	9148 (48%)	28,315 (49%)
Living with someone	8026 (42%)	25,328 (44%)
In nursing home	1395 (7%)	2233 (4%)
Unknown	533 (3%)	1430 (3%)
Individuals with ≥ 1 diagnosis within each category, *n* (%)		
Certain infections	2514 (13%)	6680 (12%)
Neoplasms	5681 (30%)	17,516 (31%)
Hematological/immunological	1632 (9%)	4437 (8%)
Endocrine/metabolic	6570 (34%)	16,630 (29%)
Mental and behavioral^*^	2772 (15%)	3692 (6%)
Nervous system*	3172 (17%)	8787 (15%)
Eye and adnexa	6604 (35%)	19,622 (34%)
Ear and mastoid process	4149 (22%)	11,206 (20%)
Circulatory system	10,837 (57%)	30,812 (54%)
Respiratory system	4027 (21%)	11,969 (21%)
Digestive system	6734 (35%)	19,583 (34%)
Skin/subcutaneous system	1852 (10%)	5408 (9%)
Musculoskeletal system	9858 (52%)	28,365 (50%)
Genitourinary system	5837 (31%)	16,149 (28%)
Symptoms/signs not classified elsewhere	12,752 (67%)	29,603 (52%)
Injuries, poisonings, external causes	10,773 (56%)	27,781 (48%)
Factors influencing health status	18,836 (99%)	52,805 (92%)
Individuals with ≥ 1 prescription within each overall category, *n* (%)		
Alimentary tract and metabolism	14,202 (74%)	39,508 (69%)
Blood and blood forming organs	12,103 (63%)	31,588 (55%)
Cardiovascular system	16,419 (86%)	45,896 (80%)
Dermatologicals	13,162 (69%)	36,701 (64%)
Genitourinary system and sex hormones	8781 (46%)	23,131 (40%)
Systemic hormonal preparations	6496 (34%)	18,935 (33%)
Antiinfectives for systemic use	17,244 (90%)	47,896 (84%)
Antineoplastic and immunomodulating agents	793 (4%)	2121 (4%)
Musculo-skeletal system	13,472 (71%)	38,017 (66%)
Nervous system*	16,389 (86%)	44,375 (77%)
Antiparasitic products, insecticides, and repellents	3730 (20%)	10,827 (19%)
Respiratory system	11,330 (59%)	32,474 (57%)
Sensory organs	12,863 (67%)	36,340 (63%)

^*^Excluding mild cognitive impairment, dementia diagnoses and antidementia medication. ^a^MMSE: Mini-Mental State Examination (reliable information for 17,648 patients). ACE: Addenbrooke’s Cognitive Examination (reliable information for 141,35 patients) Note: Where percentages do not add up to 100%, this is due to rounding up/down

### Morbidity

**Fig. 1:**
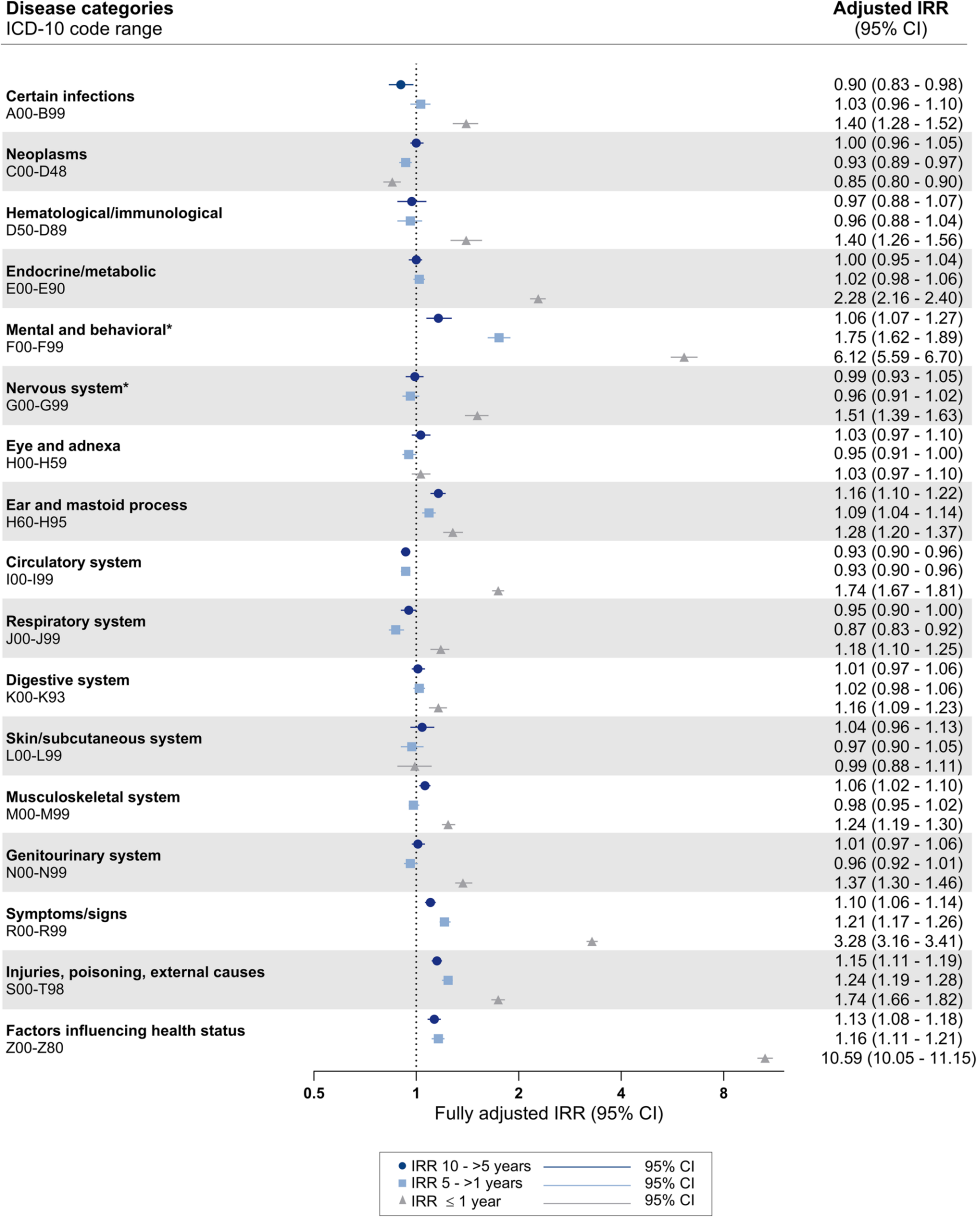
Morbidity in overall categories. Incidence rate ratios (IRRs) for late-onset Alzheimer’s disease are plotted by overall disease category in three time-intervals prior to diagnosis. For the reference group (cognitively healthy controls), the IRR is equal to 1 (indicated by the dotted vertical line). Error bars represent 95% confidence intervals (CI). The IRRs presented are adjusted for sex, age, highest attained educational level at age 50 years, and living status (living alone, living with someone, or at nursing home) at index date. *Excluding mild cognitive impairment and dementia diagnoses. ICD-10: International Classification of Diseases, 10th revision

In the year preceding the index date, statistically significantly increased IRRs were observed in 14 out of the 17 investigated disease categories, the highest three IRRs were found for Mental and behavioral disorders (6.12, 95% CI 5.59–6.70), Factors influencing health status (10.59, 95% CI 10.05–11.15), and Symptoms/signs (3.28, 95% CI 3.16–3.41). For these categories, IRRs gradually increased already from 10 to five years prior to index date. The remaining categories generally had non-significant or decreased IRRs in the time periods 10 to five years and five to one year prior to index date, except for the category Injuries, poisoning, external causes, which showed increased IRRs in all time periods (Fig. 1).

Investigating morbidity within subcategories over the 10-year period (Fig. S1) showed statistically significantly increased IRRs in 32 out of the 38 investigated subcategories, most prominently in Examination and investigation (IRR 10.00, 95% CI 8.89–11.25), Symptoms/signs involving cognition, perception, and behavior (5.49, 95% CI 5.22–5.78), and Symptoms/signs involving the nervous and musculoskeletal system (2.50, 95% CI 2.38–2.63). A few subcategories showed non-significant or decreased IRRs.

**Fig. 2:**
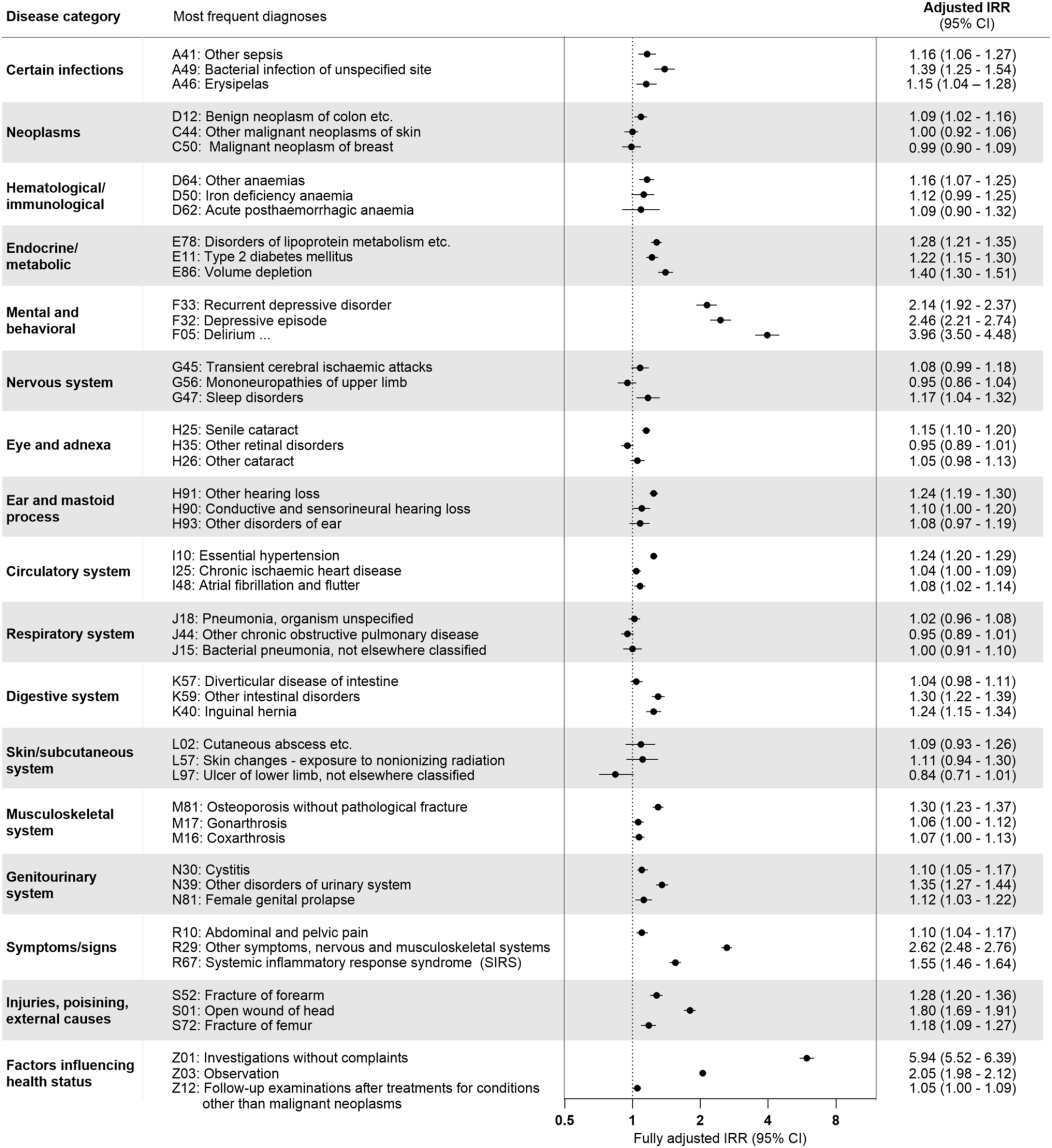
The three most frequent diagnoses within overall categories. Incidence rate ratios (IRRs) for late-onset Alzheimer’s disease are plotted for the three most frequent diagnoses within the overall disease category in the 10-year retrospective study period. For the reference group (cognitively healthy controls), the IRR is equal to 1 (indicated by the dotted vertical line). Error bars represent 95% confidence intervals (CI). The IRRs presented are adjusted for sex, age, highest attained educational level at age 50 years, and living status (living alone, living with someone, or at nursing home) at index date. ICD-10: International Classification of Diseases, 10th revision

Looking into the three most frequent diagnoses within the overall categories (Fig. 2) over the 10-year period, IRRs were statistically significantly increased in 30 out of the 51 investigated diagnoses. The largest increases were found for a number of diagnoses of unspecified symptoms, such as Investigations without complaint (IRR 5.94, 95% CI 5.52–6.39), Other symptoms, nervous and musculoskeletal systems (2.62, 95% CI 2.48–2.76), and Observation (2.05, 95% CI 1.98–2.12). The largest increases in specific diagnoses were found for Recurrent depressive disorder (IRR 2.14, 95% CI 1.92–2.37), Depressive episode (2.46, 95% CI 2.21–2.74), and Delirium (3.96, 95% CI 3.50–4.48).

### Medication use

**Fig. 3:**
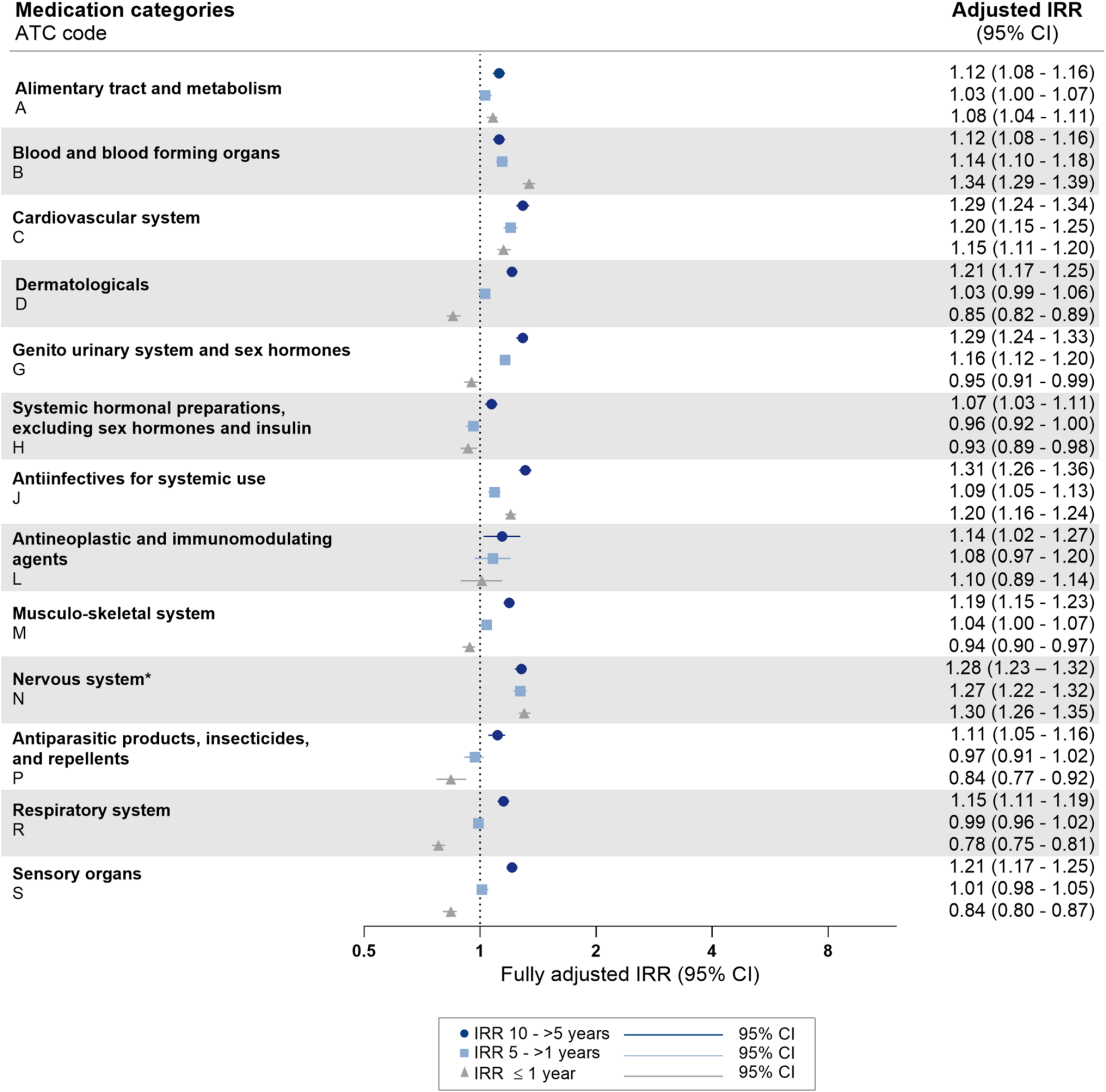
Medication use in overall categories. Incidence rate ratios (IRRs) for late-onset Alzheimer’s disease are plotted by overall medication category in three time-intervals prior to diagnosis. For the reference group (cognitively healthy controls), the IRR is equal to 1 (indicated by the dotted vertical line). Error bars represent 95% confidence intervals (CI). The IRRs presented are adjusted for sex, age, highest attained educational level at age 50 years, and living status (living alone, living with someone, or at a nursing home) at index date. *Excluding antidementia medication. ATC: Anatomical Therapeutic Chemical

In the year prior to index date, statistically significantly increased IRRs were observed in five out of the 13 investigated overall categories, while seven categories showed decreased IRRs (Fig. 3). The latter had shown increased IRRs in the period 10 to 5 years prior to index date.

The largest increases were found for Blood and blood forming organs medication (IRR 1.34, 95% CI 1.29–1.39), Nervous system medication (1.30, 95% CI 1.26–1.35), Anti-infectives for systemic use (1.20, 95% CI 1.16–1.24), and Cardiovascular system medication (1.15, 95% CI 1.11–1.20). These categories were also the only ones that showed statistically significantly increased IRRs in all time-periods. The most prominent decreases were found in Respiratory system medication (IRR 0.78, 95% CI 0.75–0.81), sensory organs medication (0.84, 95% CI 0.80–0.87), and antiparasitic products, insecticides, and repellents (0.84, 95% CI 0.77–0.92).

Figure S2 shows the association between medication subcategories and LOAD in the entire 10-year period. IRRs were statistically significantly altered in 33 out of 46 examined subcategories, with increases in 26 of these subcategories, and decreases in four. The largest increases were found in Antipsychotics (IRR 2.08, 95% CI 1.94–2.22), Antidepressants (1.87, 95% CI 1.80–1.94), and Antibacterials for systemic use (1.72, 95% CI 1.64–1.81).

Further investigating the three most frequent medications within each medication category (Fig. S3), we found statistically significantly increased IRRs in 25 out of the 39 investigated medications, most prominently in aromatase inhibitors (IRR 1.85, 95% CI 1.38–2.49), Vitamin B12 (1.64, 95% CI 1.56–1.72) and Anilides (1.32, 95% CI 1.27–1.37). IRRs were statistically significantly decreased in Pyrimidine analogues (0.60, 95% CI 0.45–0.79) and Selective beta2-adrenoreceptor agonists (0.90, 95% CI 0.87–0.94).

### Mental health morbidity and nervous system drug use

**Fig. 4:**
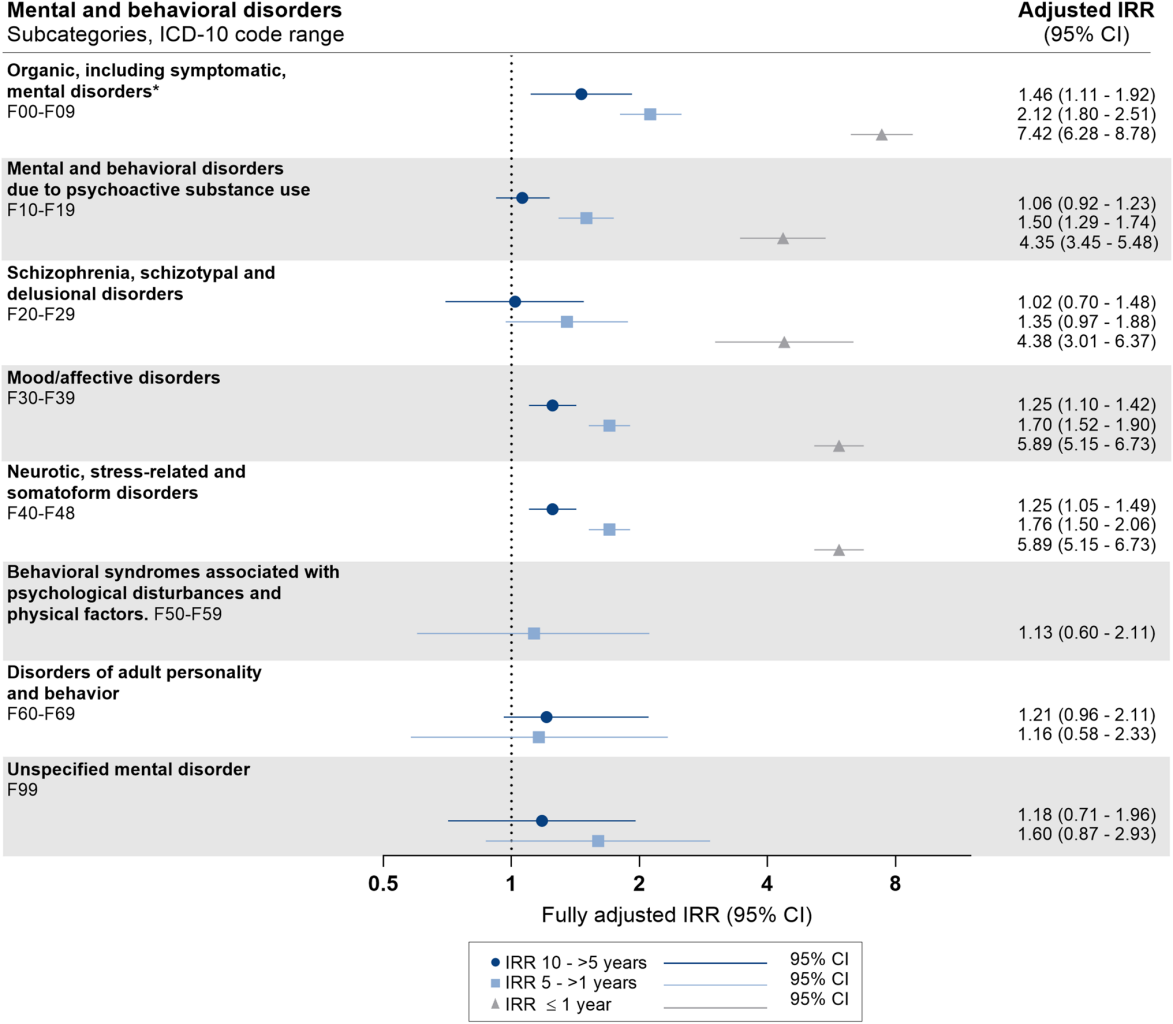
Morbidity in Mental and Behavioral disorders category. Incidence rate ratios (IRRs) for late-onset Alzheimer’s disease are plotted by subcategories of Mental and Behavioral Disorders in three time-intervals prior to diagnosis. For the reference group (cognitively healthy controls), the IRR is equal to 1 (indicated by the dotted vertical line). Error bars represent 95% confidence intervals (CI). The IRRs presented are adjusted for sex, age, highest attained educational level at age 50 years, and living status (living alone, living with someone, or at nursing home) at index date. Where no estimates are presented, there were too few events to analyze. *Excluding mild cognitive impairment and dementia diagnoses. ICD-10: International Classification of Diseases, 10th revision

Exploring the IRRs for morbidity in the Mental and Behavioral subcategory, we found statistically significantly increased IRRs in all subcategories where we were able to calculate estimates in the year prior to index. Where no estimates are presented, there were too few events to analyze. IRRs for Organic, including symptomatic mental disorders, Mood/affective disorders, and Neurotic, stress-related and somatoform disorders were statistically significantly increased in all time intervals (Fig. 4).

**Fig. 5:**
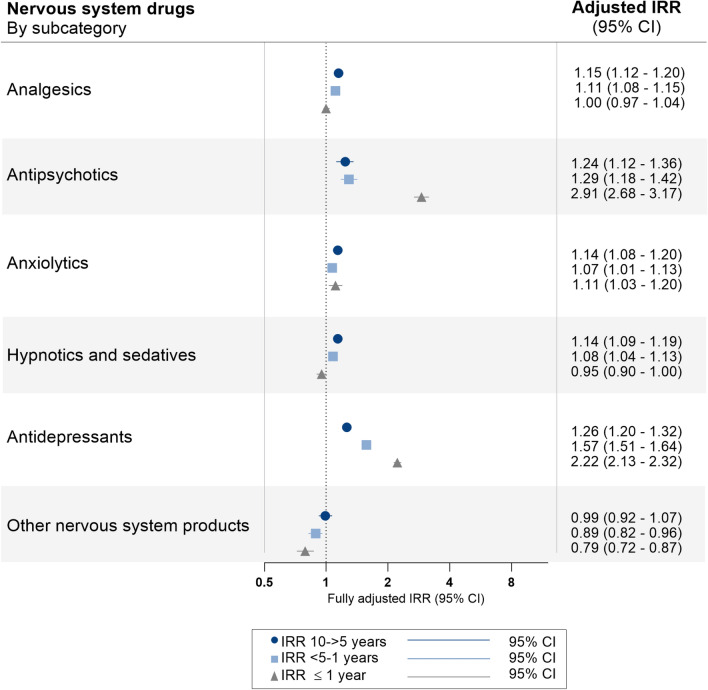
Medication use in nervous system category. Incidence rate ratios (IRRs) for late-onset Alzheimer’s disease are plotted by subcategories of Nervous System medications category in three time-intervals prior to diagnosis. For the reference group (cognitive healthy controls), the IRR is equal to 1 (indicated by the dotted vertical line). Error bars represent 95% confidence intervals (CI). The IRRs presented are adjusted for sex, age, highest attainted educational level at age 50 years, and living status (living alone, living with someone, or at nursing home) at index date. ATC: Anatomical Therapeutic Chemical

Exploring the Nervous system drugs subcategory, we found that the use of Antipsychotics and Antidepressants was statistically significantly increased in the entire 10-year period with the highest IRRs observed in the one year prior to index date (Fig. 5).

### Sensitivity analysis

Sensitivity analyses showed the same trends as the main analyses (Tables S4–S9). In a sensitivity analysis where all contacts registered during the 6 months prior to index date were censored (Tables S8–S9), allowing investigation of the period oneyear to six months prior, IRRs were attenuated but remained significantly increased. Unadjusted results are presented for the overall disease and medication categories (Tables S10–S11), demonstrating that adjustment had minimal impact on the observed associations.

## Discussion

### Summary of findings

In this nationwide nested case–control study, we compared 19,102 patients diagnosed with LOAD with 57,306 cognitively healthy, sex and age-matched controls. We found that in the earliest prodromal years, individuals later diagnosed with LOAD had more mental health and behavioral diagnoses accompanied by higher use of antipsychotics, antidepressants, sedatives and anxiolytics. Additionally, they had elevated rates of injuries, and hospital contacts with a diagnosis of unspecified symptoms/signs. As they approached a LOAD diagnosis, morbidity increased across multiple diagnostic categories, while medication use decreased in most categories.

These findings suggest that the prodromal phase of LOAD is characterized by gradual accumulation of morbidity with neuropsychiatric symptoms as the most persistent throughout the entire period. This is not only a great cause of distress for the patients and caregivers but may also contribute to the increased hospital contacts regarding injuries and non-specific symptoms. Importantly, our findings show that these individuals are already in contact with healthcare professionals during this prodromal phase, providing potential for recognizing early warning signs.

### Interpretation and comparison to previous literature

Previous research has demonstrated that the years preceding a diagnosis of dementia or AD are characterized by increasing morbidity; particularly involving neuropsychiatric conditions, injuries, gait abnormalities, dizziness, and sensory impairments [17–22]. However, the temporal development of these changes and corresponding medication patterns as demonstrated in this study has been lacking.

Consistent with our earlier findings in young-onset Alzheimer’s disease [30, 31], we found that the early prodromal phase is characterized by increasing rates of mental health-related conditions, injuries, and non-specific healthcare contacts without a clear underlying diagnosis.

We found these increases up to 10 years before diagnosis and increased across the observation period. Their timing, gradual progression, and alignment with known AD-related neuropathological changes [40, 41] support the interpretation that these features represent prodromal features.

Neuropsychiatric symptoms were observed as some of the earliest signs; therefore, we investigated whether these symptoms emerged in the 10-year prodromal phase or reflected pre-existing conditions. In a post-hoc review of the data, we identified 6244 individuals among cases and controls who received a diagnosis within the mental and behavioral category during the 10-year study period. Of these, only 1262 (449 cases) had a recorded mental and behavioral diagnosis prior to the study period. This suggests that neuropsychiatric symptoms emerged within the observation period, supporting that such symptoms may represent prodromal features and are among the earliest signs.

The literature contains extensive evidence linking neuropsychiatric symptoms to early AD pathology but also showing that these symptoms may accelerate cognitive decline [42]. For depression specifically, previous studies identify both its role as a risk factor and as a prodromal symptom[43]. Given shared biological mechanisms—such as hippocampal atrophy and neuroinflammation—the relationship is likely bidirectional, and similar bidirectionality may underlie other associations identified in our study, such as increased rates of infections and injuries, which are discussed in the next sections.

Injuries emerged as a prominent prodromal feature. Individuals later diagnosed with LOAD had higher rates of injuries as early as 10 years before diagnosis. Prior studies have shown that individuals with AD have more than twice the risk of falls than cognitively healthy adults [41], a vulnerability attributed to impaired executive function, altered gait and balance, and polypharmacy, particularly with psychopharmaceuticals. While an increased injury rate likely reflects early cognitive and functional decline, it is also notable that falls, fractures, and head injuries themselves may accelerate or unmask underlying pathology, potentially contributing to disease progression.

We found an increase in hospital contacts coded under the ICD-10 category “Factors influencing health status” with the three most frequent diagnoses being examination and investigation, investigations without complaints, and observation. This likely reflects a patient population presenting with vague or non-specific symptoms, or subtle cognitive impairment that complicates treatment at home. Together, these findings depict a gradual rise in clinical vulnerability and diagnostic uncertainty long before a formal diagnosis is established.

In the years immediately preceding diagnosis, we observed increasing morbidity along with decreasing medication use in most categories. This pattern may reflect growing vulnerability leading to more hospital encounters and potentially leading to a clinical review and discontinuation of non-essential medications. Alternatively, it may indicate declining medication adherence as cognitive decline progresses, coupled with reduced symptom awareness and delayed healthcare seeking, ultimately resulting in hospitalization. Both explanations are plausible, and together they likely represent broader physiological and cognitive decline. Our study found associations between LOAD and several well-established risk factors[44], including cardiovascular diseases, diabetes mellitus, and hearing loss.

We further observed a higher rate of infections and lower rate of malignant neoplasms in patients later diagnosed with LOAD. The use of systemic anti-infectives was increased throughout the entire 10-year period. However, hospital contacts coded with the ICD-10 code “certain infections” – including pathogen specific infections such as erysipelas and sepsis—as well as other categories including common infections such as respiratory and genitourinary infections were only increased in the year immediately preceding diagnosis. This likely reflects worsening overall health in the late prodromal stage, or difficulties in recognizing infections early enough to be treated in primary care as patients may not report symptoms. A previous Danish study has also reported increased infection-related hospital contacts in the five years prior to dementia diagnosis and increasing closer to diagnosis [45]. Other previous studies found an increased long-term risk of dementia following hospitalization with bacterial and other infections [46–48]. The mechanisms linking infections to dementia remain unclear, but suggested ones include that systemic inflammation is associated with risk of developing dementia because of immunological and inflammatory changes, or a weakened immune system may predispose to both higher infections rates and the development of dementia. The relationship is likely also bidirectional, and patients later developing LOAD are probably more vulnerable to infections, but infections may also cause, accelerate, or unmask dementia pathology [49].

Finally, we observed lower rates of malignant neoplasms prior to LOAD. However, this was accompanied by increased use of antineoplastic and immunomodulating agents during the 10–five years preceding diagnosis, as well as an elevated use of aromatase inhibitors when looking at the entire 10-year period. A negative association between malignant neoplasms and dementia has been reported repeatedly, and several explanations have been proposed [50, 51]. One possibility is reduced diagnostic evaluation; older, cognitively impaired patients may be less likely to report symptoms, and clinicians may be less likely to pursue extensive diagnostic evaluation, especially given that a good overall health is typically required for cancer treatment. Another explanation could be that individuals diagnosed with advanced cancer are more likely to get diagnosed with dementia in general practice and not be referred for more extensive evaluation in memory clinics, thus not entering our study population.

The increased use of aromatase inhibitors is notable, especially since breast cancer itself was not significantly associated with later LOAD. This likely reflects prior breast cancer diagnosis occurring before our 10-year observation window, while ongoing aromatase inhibitor therapy–typically continued for five years–was still captured in our study. Another explanation relates to the debated role of aromatase inhibitors in dementia risk: existing studies show mixed findings, ranging from no association or reduced risk to a possible increased risk [52, 53].

### Methodological considerations

Our study has several strengths. Data were drawn from high quality Danish registers, providing comprehensive information on morbidity, medication use, and relevant covariates for the entire Danish population. This nationwide coverage ensured that all eligible Danish citizens could serve as controls, thereby minimizing selection bias and enabling longitudinal follow-up. Cases were identified from DanDem, which ensured high diagnostic validity for LOAD diagnosis and included all patients diagnosed at Danish memory clinics. Examining outcomes across different time periods provided insights into temporal patterns. To our knowledge, this is the first study to describe such patterns, addressing a gap in knowledge regarding early signs of LOAD, and strengthening the characterization of the prodromal phase.

However, certain limitations should be acknowledged. The DanDem register does not include diagnoses made outside of memory clinics, meaning that cases identified only by general practitioners or in hospital care are not included. Further, we do not include all registered AD patients in our study. As shown in a previous study [48], approximately one-third of registered dementia cases are diagnosed outside of memory clinics. The same study demonstrated that the median age of those not diagnosed in a memory clinic was higher at diagnosis, they had more comorbidities and a higher mortality within one year of a registered diagnosis. Thus, the registered dementia cases, many likely AD cases, that we did not include were probably patients in advanced disease stages.

It is also important to note that with increasing registered morbidity, contact with healthcare professionals is more frequent and the likelihood of referral to a memory clinic will increase, as this increases the likelihood that a healthcare professional notices symptoms of dementia and refers the patient. Regarding prior morbidity mapping, diseases managed exclusively in primary care were not captured; however, these were represented if they received pharmacological treatment as data on all redeemed medications were available. Finally, the validity of some of the recorded diagnostic codes is unknown, which may affect the precision of the observed associations. The external validity of this study is unknown, so it is unclear to what extent our results are influenced by the Danish social and healthcare systems, and whether similar patterns would be observed in other populations.

## Conclusions

In conclusion, individuals later diagnosed with LOAD had more mental health and behavioral diagnoses accompanied by higher use of antipsychotics, antidepressants, sedatives and anxiolytics in the earliest prodromal years. Additionally, they had more injuries, and hospital contacts with a diagnosis of unspecified symptoms/signs. As diagnosis approached, morbidity increased across multiple diagnostic categories, while medication use decreased in most categories. These findings can guide future hypothesis-driven studies to better characterize the prodromal phase which may improve early detection, reduce the burden of unexplained symptoms experienced by patients and their caregivers, and support clinicians facing diagnostic uncertainty.

To our knowledge, this is the first study of the prodromal phase to provide a broad overview of hospital contacts and medicine changes prior to diagnosis. However, further research is needed to explore the specific clinical presentations underlying these contacts.

## Supplementary Information

Below is the link to the electronic supplementary material.

Supplementary file1 (DOCX 1479 KB)

## Data Availability

All data used in this study are derived from the Danish National and Public Health registries. These data are collected and stored by the relevant authorities and cannot be made public or accessed by unauthorized parties. Access to such data is given via standard rules and regulations of data access outlined by the Danish Data Protection Agency and Danish Health Data Authority.
