# Supplementary material for: Morbidity and medication use preceding a diagnosis of late-onset Alzheimer’s disease: a Danish nationwide study
Source: J Neurol. 2026 Jul 2;273(8):443. doi: 10.1007/s00415-026-13967-y (PMC13328233; doi:10.1007/s00415-026-13967-y)
Supplement: Supplementary file 1 — Supplementary file1 (DOCX 1479 KB) [file 415_2026_13967_MOESM1_ESM.docx]

### Supplementary tables and figures

**Morbidity and medication use preceding a diagnosis of late-onset Alzheimer’s disease: A Danish nationwide study**

**Journal of Neurology**

**Authors:** Cecilia El-Sayed Petersen^1*^, Line Damsgaard^1*^, Janet Janbek^1^, Thomas Munk Laursen^2^, Karsten Vestergaard^3^, Hanne Gottrup^4^, Gunhild Waldemar^1,5^

Cecilia El-Sayed Petersen, MSc^1^

^1^Danish Dementia Research Centre, Section 8008, Department of Neurology, Copenhagen University Hospital - Rigshospitalet, Copenhagen, Denmark

Address: Blegdamsvej 9, 2100 Copenhagen, Denmark

Telephone: +45 42 17 14 74

Email: [Cecilia.el-sayed.petersen@regionh.dk](mailto:Cecilia.el-sayed.petersen@regionh.dk)

Line Damsgaard, MD, PhD^1^

^1^Danish Dementia Research Centre, Section 8008, Department of Neurology, Copenhagen University Hospital - Rigshospitalet, Copenhagen, Denmark

Address: Blegdamsvej 9, 2100 Copenhagen, Denmark

Telephone: +45 28 44 59 22

Email: [line.damsgaard@regionh.dk](mailto:line.damsgaard@regionh.dk)

Janet Janbek, PhD^1^

^1^Danish Dementia Research Centre, Section 8008, Department of Neurology, Copenhagen University Hospital - Rigshospitalet, Copenhagen, Denmark

Address: Blegdamsvej 9, 2100 Copenhagen, Denmark

Email: [janet.janbek@regionh.dk](mailto:janet.janbek@regionh.dk)

Thomas Munk Laursen, PhD^2^

^2^National Centre for Register-based Research, Department of Economics and Business Economics, Aarhus BSS, Aarhus University, Aarhus, Denmark
Address: Fuglesangs Allé 26- Building R, 8210 Aarhus V, Denmark

Email: [tml.ncrr@au.dk](mailto:tml.ncrr@au.dk)

Karsten Vestergaard^3^, MD

^3^Dementia Clinic, Department of Neurology, Aalborg University Hospital, Aalborg, Denmark

Address: Ladegaardsgade 5, 9000 Aalborg, Denmark

Email: [k.vestergaard@rn.dk](mailto:k.vestergaard@rn.dk)

Hanne Gottrup, MD, PhD^4^

^4^Dementia Clinic, Department of Neurology, Aarhus University Hospital, Aarhus, Denmark

Address: Palle Juul-Jensen Boulevard 99, 8200 Aarhus N, Denmark

Email: [hanngott@rm.dk](mailto:hanngott@rm.dk)

Gunhild Waldemar, MD, DMSc^1,5^

^1^Danish Dementia Research Centre, Section 8008, Department of Neurology, Copenhagen University Hospital - Rigshospitalet, Copenhagen, Denmark

Address: Blegdamsvej 9, 2100 Copenhagen, Denmark
^5^Department of Clinical Medicine, University of Copenhagen

Address: Blegdamsvej 3B, 2200 Copenhagen, Denmark

Email: [gunhild.waldemar.01@regionh.dk](mailto:gunhild.waldemar.01@regionh.dk)

*Shared first authorship

**Correspondence:** Janet Janbek, PhD

Danish Dementia Research Centre, Section 8008, Department of Neurology, Copenhagen University Hospital - Rigshospitalet, Copenhagen, Denmark

Address: Blegdamsvej 9, 2100 Copenhagen, Denmark

Email: [janet.janbek@regionh.dk](mailto:janet.janbek@regionh.dk)

**Contents:**

**Table S1.** Exclusion criteria

**Table S2.** Overall morbidity categories, subcategories, and corresponding ICD-10 codes

**Table S3.** Overall medication categories, subcategories, and corresponding ATC codes

**Figure S1.** Incidence rate ratios by disease subcategories over a 10-year period

**Figure S2** Incidence rate ratios by medication subcategories over a 10-year period

**Figure S3.** The three most frequent medications within each medication overall category and corresponding incidence rate ratios over a 10-year period

**Table S4.** Sensitivity analysis by dementia syndrome severity at time of diagnosis – incidence rate ratio by disease category in three time-intervals

**Table S5.** Sensitivity analysis by dementia syndrome severity at time of diagnosis – incidence rate ratio by medication category in three time-intervals

**Table S6** Sensitivity analysis by sex and age – incidence rate ratio by disease category in three time-intervals

**Table S7.** Sensitivity analysis by sex and age – incidence rate ratio by medication category in three time-intervals

**Table S8.** Sensitivity analysis censoring contacts 6 months before index date – Incidence rate ratios by disease category in the time-interval <1- 6 months prior to diagnosis of late-onset Alzheimer’s disease

**Table S9.** Sensitivity analysis censoring contacts 6 months before index date – Incidence rate ratios by medication category in the time-interval <1- 6 months prior to diagnosis of late-onset Alzheimer’s disease

**Table S10**. Main analysis – unadjusted incidence rate ratios by overall disease categories in three time-intervals

**Table S11.** Main analysis – unadjusted incidence rate ratios by overall medication categories in three timer-intervals

**Table S1. Exclusion criteria**

|  | Cases | Controls |
| --- | --- | --- |
| Dementia diagnosis  ICD-8: 290.09-11, 290.18-19, 293.09-19, ICD 10: F00.0-00.9, F01.0-01.9, F02.0-F02.8, F03.9, G30.0-G30.9, G31.0A, G31.0B, G31.8, G31.8E, G31.9 | * | X |
| Dementia medication  ATC code N06DA02-4, N06DX01 | ** | X |
| Entry in DanDem |  | X |

* These ICD10 codes registered in Danish National Patient Register and the Danish Psychiatric Central Research Register are omitted in the analyses.

** These ATC codes registered in the Danish National Prescription Registry are omitted in the analyses.

ICD: International Classification of Diseases, ATC: Anatomical Therapeutic Chemical code, DanDem: Danish Quality Database for Dementia

**Table S2.** Overall morbidity categories, subcategories, and corresponding ICD-10 codes

| Overall category and ICD-10 code range | Subcategory | ICD-10 codes |
| --- | --- | --- |
| Certain infections  A00-B99 | *Intestinal infectious diseases‡* | *A00-A09‡* |
|  | *Viral infections of the central nervous system‡* | *A80-A89 ‡* |
|  | *Viral infections characterized by skin and mucous membrane lesions‡* | *B00-B09‡* |
|  | *Chronic viral infections (Hepatitis, HIV)‡* | *B15-19, B20-24‡* |
|  | *Other infectious diseases‡* | *A15-A19, A20-28, A30-49, A50-A64, A70-A74, A75-A79, A92-A99, B25-B34, B50-B64, B65-B83, B85-B89, B90-B94, B95-B98, B99‡* |
| Neoplasms  C00-D48 | Malignant neoplasms | C00-C97 |
|  | *In situ neoplasms** | *D00-D09** |
|  | Benign Neoplasms | D10-D36 |
|  | *Neoplasms of uncertain or unknown behaviour** | *D37-D48** |
| Hematological/immunological diseases  D50-D89 | Anaemias | D50-53, D55-D59, D60-D64 |
|  | *Coagulation defects, purpura and other haemorrhagic conditions** | *D65-D69** |
|  | *Other diseases of blood and blood-forming organs†* | *D65-D69, D70-D77, D80-D89†* |
| Endocrine/metabolic diseases  E00-E90 | Disorders of the thyroid gland | E00-E07 |
|  | Diabetes mellitus | E10-E14 |
|  | *Obesity and other hyperalimentation* | *E65-E68** |
|  | Other endocrine, nutritional and metabolic diseases | E15-E16, E20-E35, E40-E46, E50-E64, E65-E68, E70-E90 |
| Mental and behavioral disorders  F00-F99 | *Organic, including symptomatic, mental disorders‡* | *F00-F09‡* |
|  | *Mental and behavioural disorders due to psychoactive substance use‡* | *F10-F19‡* |
|  | *Schizophrenia, schizotypal and delusional disorders‡* | *F20-F29‡* |
|  | *Mood [affective] disorders‡* | *F30-F39‡* |
|  | *Neurotic, stress-related and somatoform disorders‡* | *F40-F48‡* |
|  | *Behavioural syndromes associated with physiological disturbances and physical factors‡* | *F50-F59‡* |
|  | *Disorders of adult personality and behaviour‡* | *F60-F69‡* |
|  | *Mental retardation and early onset psychiatric disorders‡* | *F70-F79, F80-F89, F90-F98‡* |
|  | *Unspecified mental disorder‡* | *F99-F99‡* |
| Diseases of the nervous system  G00-G99 | *Inflammatory diseases of the central nervous system** | *G00-G09** |
|  | *Systemic atrophies primarily affecting the central nervous system** | *G10-G14** |
|  | *Extrapyramidal and movement disorders** | *G20-G26** |
|  | *Other degenerative diseases of the nervous system [excluding dementia diagnoses]** | *G30-G32** |
|  | *Demyelinating diseases of the central nervous system** | *G35-G37** |
|  | Episodic and paroxysmal disorders | G40-G47 |
|  | *Nerve, nerve root and plexus disorders** | *G50-G59** |
|  | *Polyneuropathies and other disorders of the peripheral nervous system** | *G60-G64** |
|  | Other diseases of the nervous system | G00-G09, G10-G14, G20-G26, G30-G32, G35-G37, G50-G59, G60-G64, G70-G73, G80-G83, G90-G99 |
| Diseases of the eye and adnexa  H00-H59 | Glaucoma‡ | *H40-H42*‡ |
|  | *Disorders of ocular muscles, binocular movement, accommodation and refraction*‡ | *H49-H52*‡ |
|  | *Visual disturbances and blindness*‡ | *H53-H54*‡ |
|  | *Other disorders of the eye and visual system*‡ | *H00-H06, H10-H13, H15-H22, H25-H28, H30-H36, H43-H45, H46-H48, H55-H59*‡ |
| Diseases of the ear and mastoid process  H60-H95 | *Diseases of external ear‡* | *H60-H62‡* |
|  | *Diseases of middle ear and mastoid‡* | *H65-H75‡* |
|  | *Diseases of inner ear‡* | *H80-H83‡* |
|  | *Other disorders of ear‡* | *H90-H95‡* |
| Diseases of the circulatory system  I00-I99 | Hypertensive diseases | I10-I15 |
|  | Ischaemic heart diseases | I20-I25 |
|  | Cerebrovascular diseases | I60-I69 |
|  | Diseases of arteries, arterioles and capillaries | I70-I79 |
|  | Diseases of veins, lymphatic vessels and lymph nodes, not elsewhere classified | I80-I89 |
|  | Other circulatory system disorders | I00-I02, I05-I09, I26-I28, I30-I52, I95-I99 |
| Diseases of the respiratory system  J00-J99 | *Acute upper respiratory infections** | *J00-J06** |
|  | *Influenza and pneumonia** | *J09-J18** |
|  | Chronic lower respiratory diseases | J40-J47 |
|  | Other diseases of the respiratory system | J00-J06, J09-J18, J20-J22, J30-J39, J60-J70, J80-J84, J85-J86, J90-J94, J95-J99 |
| Diseases of the digestive system  K00-K93 | Diseases of oesophagus, stomach and duodenum | K20-K31 |
|  | *Diseases of appendix** | *K35-K38** |
|  | Hernia | K40-K46 |
|  | *Noninfective enteritis and colitis** | *K50-K52** |
|  | *Diseases of peritoneum** | *K65-K67** |
|  | *Diseases of liver** | *K70-K77** |
|  | *Disorders of gallbladder, biliary tract and pancreas** | *K80-K87** |
|  | Other digestive system disorders | K00-K14, K35-K38, K50-K52, K55-K64, K65-K67, K70-K77, K80-K87, K90-K93 |
| Diseases of the skin/subcutaneous system  L00-L99 | *Infections of the skin and subcutaneous tissue‡* | *L00-L08‡* |
|  | *Bullous disorders‡* | *L10-L14‡* |
|  | *Dermatitis and eczema‡* | *L20-L30‡* |
|  | *Urticaria and erythema‡* | *L50-L54‡* |
|  | *Other skin and subcutaneous tissue disorders‡* | *L40-L45, L55-L59, L60-L75, L80-L99‡* |
| Diseases of the musculoskeletal system  M00-M99 | Arthropathies | M00-M25 |
|  | *Systemic connective tissue disorders** | *M30-M36** |
|  | Soft tissue disorders | M60-M79 |
|  | *Osteopathies and chondropathies** | *M80-M94** |
|  | Other musculoskeletal system and connective tissue disorders | M30-M36, M80-M94, M40-M54, M95-M99 |
| Diseases of the genitourinary system  N00-N99 | *Glomerular diseases** | *N00-N08** |
|  | *Renal tubulo-interstitial diseases** | *N10-N16** |
|  | *Renal failure** | *N17-N19** |
|  | *Urolithiasis** | *N20-N23** |
|  | Other kidney, ureter, and urinary system disorders | N25-N29, N30-N39 |
|  | Diseases of male genital organs | N40-N51 |
|  | *Disorders of breast** | *N60-N64** |
|  | *Inflammatory diseases of female pelvic organs** | *N70-N77** |
|  | Noninflammatory disorders of female genital tract | N80-N98 |
|  | *Other disorders of the genitourinary system†* | *N00-N08, N10-N16, N17-N19, N20-N23, N60-N64, N70-N77, N99-N99†* |
| Symptoms/signs not classified elsewhere  R00-R99 | Symptoms and signs involving the circulatory and respiratory systems | R00-R09 |
|  | Symptoms and signs involving the digestive system and abdomen | R10-R19 |
|  | *Symptoms and signs involving the skin and subcutaneous tissue** | *R20-R23** |
|  | Symptoms and signs involving the nervous and musculoskeletal systems | R25-R29 |
|  | Symptoms and signs involving the urinary system | R30-R39 |
|  | Symptoms and signs involving cognition, perception, emotional state and behaviour | R40-R46 |
|  | *Symptoms and signs involving speech and voice** | *R47-R49** |
|  | Abnormal findings on examination, without diagnosis | R70-R79,R80-R82,R83-R89,R90-R94 |
|  | *Ill-defined and unknown causes of mortality** | *R95-R99** |
|  | Other symptoms and signs | R20-R23, R47-R49, R50-R69, R95-R99 |
| Injuries, poisoning, and other external causes  S00-T98 | Injuries to the head, neck, and thorax | S00-S09, S10-S19, S20-S29 |
|  | *Injuries to the abdomen, lower back, lumbar spine and pelvis** | *S30-S39** |
|  | Injuries of the upper extremity | S40-49, S50-59, S60-69 |
|  | Injuries of the lower extremity | S70-79, S80-S89, S90-S99 |
|  | *Burns and corrosions** | *T20-T32** |
|  | *Poisonings and toxic effects** | *T36-T50, T51-T65** |
|  | Other injuries and sequelae | S30-S39, T00-T07, T08-T14, T15-T19, T20-T32, T33-T35, T36-T50, T51-T65, T66-T78, T79, T80-T88, T90-T98 |
| Factors influencing health status etc  Z00-Z80 | Persons encountering health services for examination and investigation | Z00-Z13 |
|  | *Persons with potential health hazards related to communicable diseases** | *Z20-Z29** |
|  | *Persons encountering health services in circumstances related to reproduction** | *Z30-Z39** |
|  | Persons encountering health services for specific procedures and health care | Z40-Z54 |
|  | *Persons with potential health hazards related to socioeconomic and psychosocial circumstances** | *Z55-Z65** |
|  | Persons encountering health services in other circumstances | Z70-Z76 |
|  | Persons with potential health hazards related to family and personal history and certain conditions influencing health status | Z80-Z99 |
|  | *Other factors influencing health status†* | *Z20-Z29, Z30-Z39, Z55-Z65†* |

Subcategories in cursive were not individually analyzed:

* These categories were proposed as individual categories, but due to <5% of the study population having at least one redeemed prescription during the study period, these subcategories were combined with the “other” category within that overall category.

† As there were <5% of the study population with a redeemed prescription in the proposed “other” category, this was omitted from the analysis.

‡ Within the overall categories Certain infections, Mental and behavioral disorders, Diseases of the eye and adnexa, Diseases of the ear and mastoid process, and Diseases of the skin/subcutaneous system there were no proposed subcategories where >5% of the study population had a redeemed prescription. Therefore, there were no relevant subcategories to examine for these overall categories, as combined “other” categories of all subcategories would mirror the analysis of the overall category.

**Table S3.** Overall medication categories, subcategories, and corresponding ATC codes

| Overall category and ATC code | Subcategory | ATC codes |
| --- | --- | --- |
| Alimentary tract and metabolism | Drugs for acid related disorders | A02 |
| A | *Antiemetics and antinauseants** | *A04** |
|  | Drugs for constipation | A06 |
|  | Antidiarrheals, intestinal ntologicalatory/antiinfective agents | A07 |
|  | *Antiobesity preparations, excl. diet products** | *A08** |
|  | Digestives, vitamins and mineral supplements, tonics | A09, A11, A12, A13 |
|  | Drugs used in diabetes | A10 |
|  | Other alimentary tract and metabolism products | A01, A03, A04, A05, A08, A14, A15, A16 |
| Blood and blood forming organs | Antithrombotic agents | B01 |
| B | *Antihemorrhagics** | *B02** |
|  | Antianemic preparations | B03 |
|  | *Other hematological agents†* | *B01, B02, B05, B06†* |
| Cardiovascular system | Cardiac therapy | C01 |
| C | Antihypertensives | C02, C08, C09 |
|  | Diuretics | C03 |
|  | Beta blocking agents | C07 |
|  | Other cardiovascular system products | C04, C05 |
| Dermatologicals | *Antipruritics, incl. antihistamines, anesthetics, etc., antipsoriasis* | *D04, D05** |
| D | Antibiotics and chemotherapeutics for dermatological use | D06 |
|  | Corticosteroids, dermatological preparations | D07 |
|  | *Anti-acne preparations** | *D10** |
|  | Other dermatological products | D01, D02, D03, D04, D05, D08, D09, D10, D11 |
| Genito urinary system and sex hormones | *Gynecological antiinfectives and antiseptics** | *G01** |
| G | Sex hormones and modulators of the genital system | G03 |
|  | Urologicals | G04 |
|  | Other gynecologicals | G01, G02 |
| Systemic hormonal preparations, excluding sex hormones and insulin  H | *Pituitary and hypothalamic hormones and analogues** | *H01** |
|  | Corticosteroids for systemic use | H02 |
|  | Thyroid therapy | H03 |
|  | *Pancreatic hormones** | *H04** |
|  | *Calcium homeostasis** | *H05** |
|  | *Other systemic hormonal preparations* | *H01, H04, H05†* |
| Antiinfectives for systemic use | Antibacterials for systemic use | J01 |
| J | Antimycotics for systemic use | J02 |
|  | Antivirals for systemic use | J05 |
|  | Vaccines | J07 |
|  | *Other antiinfective products†* | *J04, J06†* |
| Antineoplastic and immunomodulating agents | *Antieneoplastic agents‡* | *L01‡* |
|  | *Endocrine therapy‡* | *L02‡* |
| L | *Immunostimulants‡* | *L03‡* |
|  | *Immunosuppressants‡* | *L04‡* |
| Musculo-skeletal system | Antiinflammatory and antirheumatic products | M01 |
| M | *Muscle relaxants** | *M03** |
|  | Antigout preparations | M04 |
|  | Drugs for treatment of bone diseases | M05 |
|  | Other ntolog-skeletal products | M02, M03, M09 |
| Nervous system | Analgesics | N02 |
| N | *Antiepileptics** | *N03** |
|  | *Anti-parkinson drugs** | *N04** |
|  | Antipsychotics | N05A |
|  | Anxiolytics | N05B |
|  | Hypnotics and sedatives | N05C |
|  | Antidepressants | N06A, N06B |
|  | Other nervous system products | N01, N03, N04, N07, N06C |
| Antiparasitic products, insecticides, and repellents  P | *None chosen ‡* | *‡* |
| Respiratory system | Nasal preparations | R01 |
| R | *Throat preparations** | *R02** |
|  | Drugs for obstructive airway diseases | R03 |
|  | Antihistamines for systemic use | R06 |
|  | Other respiratory system products | R02, R05, R07 |
| Sensory organs | Ophthalmologicals | S01 |
| S | Otologicals | S02 |
|  | Ophthalmologicals and ntological preparations | S03 |

Subcategories in cursive were not individually analyzed:

* These categories were proposed as individual categories, but due to <5% of the study population having at least one redeemed prescription during the study period, these subcategories were combined with the “other” category within that overall category.

† As there were <5% of the study population with a redeemed prescription in the proposed “other” category, this was omitted from the analysis.

‡ Within the overall category Antineoplastic and immunomodulating agents, there were no proposed subcategories where >5% of the study population had a redeemed prescription. Within the Antiparasitic products, insecticides and repellents no relevant subcategories were proposed. Therefore, there were no relevant subcategories to examine for these two overall categories.

The ATC main group “Various” were omitted from all analyses due to low number of observations.

**Figure S1. Incidence rate ratios by morbidity subcategories over a 10-year period**


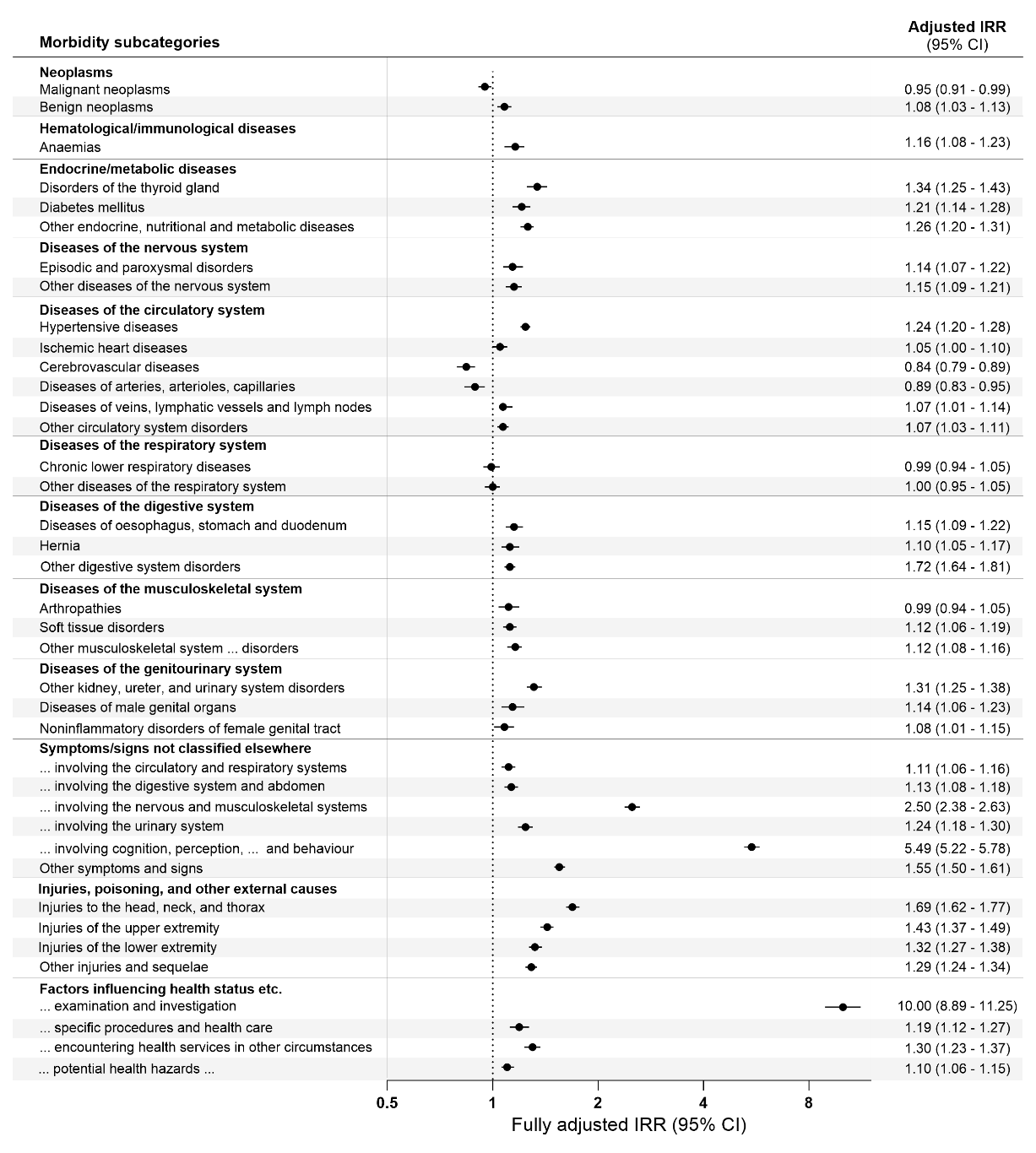
Incidence rate ratios (IRRs) for late onset Alzheimer’s disease are plotted by morbidity subcategories in the 10-year retrospective study period. For the reference group (cognitively healthy controls), the IRR is equal to 1 (indicated by the dotted vertical line). Error bars represent 95% confidence intervals (CI). The IRRs presented are adjusted for sex, age, highest attainted educational level at age 50 years, and living status (living alone, living with someone, or at nursing home) at index date.

*Excluding mild cognitive impairment and dementia diagnoses.

**Figure S2.** **Incidence rate ratios by medication subcategories over a 10-year period**


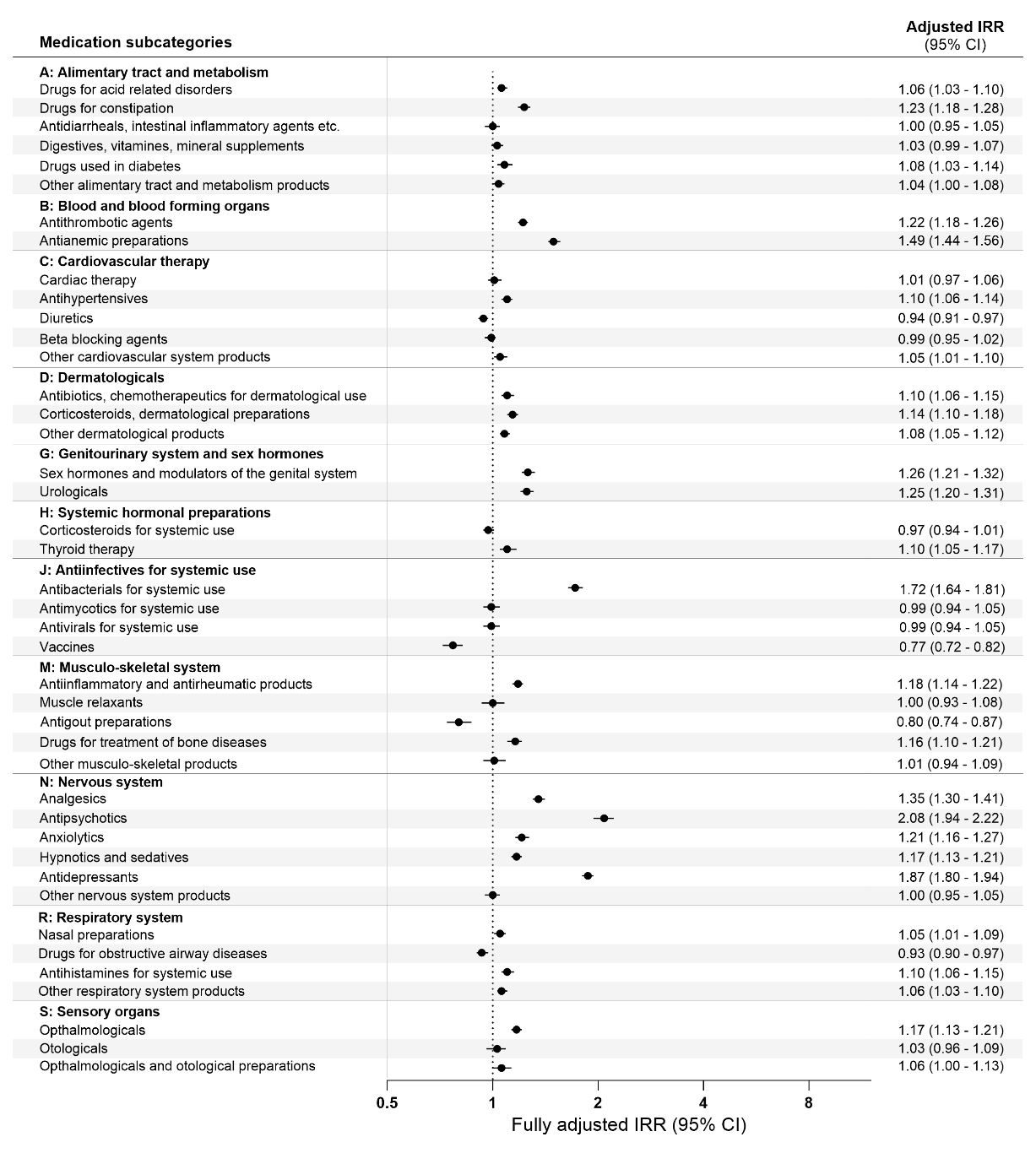


Incidence rate ratios (IRRs) for late onset Alzheimer’s disease are plotted by ATC medication subcategories in the 10-year retrospective period. For the reference group (cognitively healthy controls), the IRR is equal to 1 (indicated by the dotted vertical line). Error bars represent 95% confidence intervals (CI). The IRRs presented are adjusted for sex, age, highest attainted educational level at age 50 years, and living status (living alone, living with someone, or at nursing home) at index date.

*Excluding anti dementia medication. ATC: Anatomical Therapeutic Chemical.

**Figure S3.** **The three most frequent medications within each medication category and corresponding incidence rate ratios over a 10-year period**


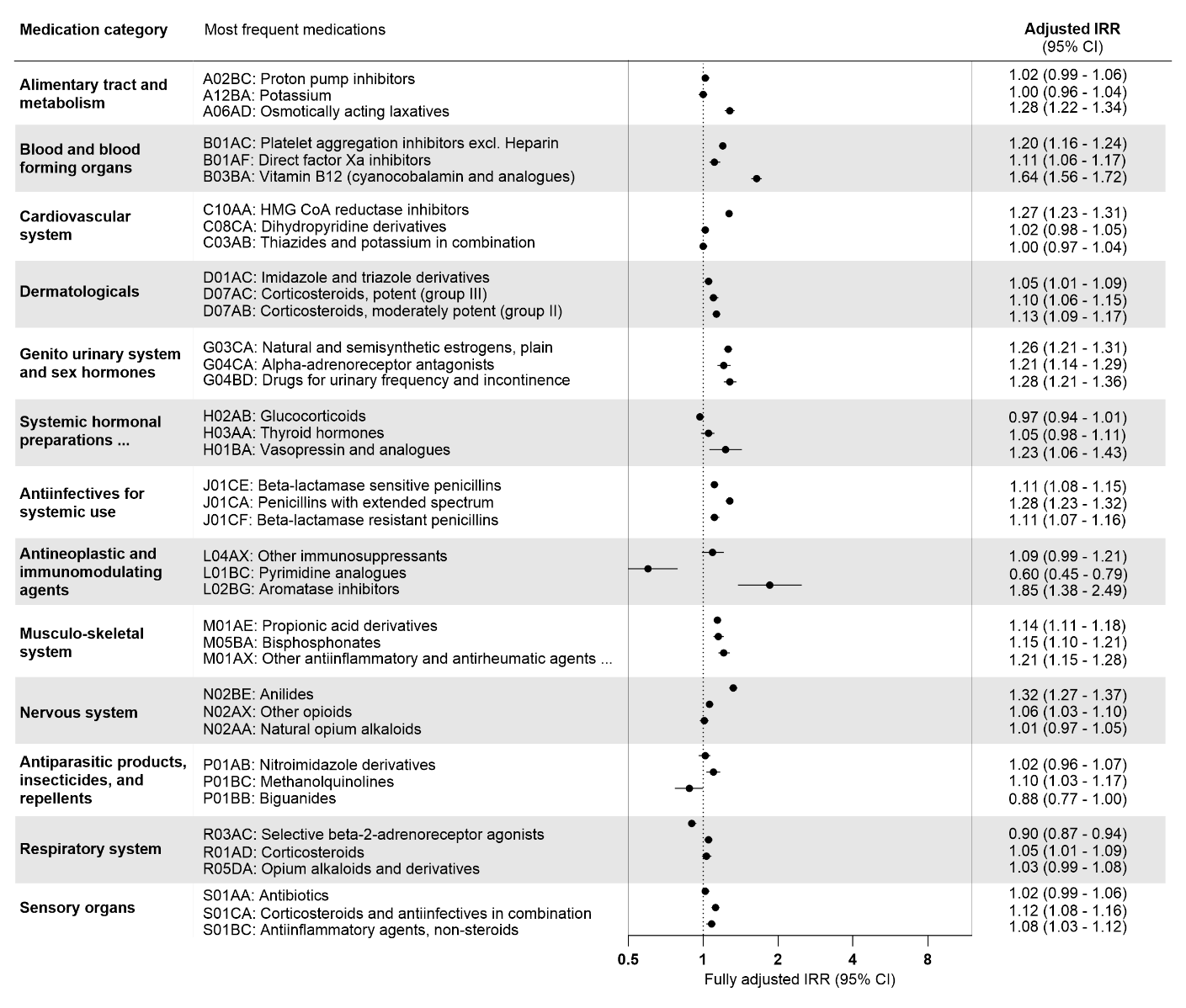


Incidence rate ratios (IRRs) for late onset Alzheimer’s disease are plotted by most frequent medication within ATC medication subcategories in the 10-year retrospective period. For the reference group (healthy controls), the IRR is equal to 1 (indicated by the dotted vertical line). Error bars represent 95% confidence intervals (CI). The IRRs presented are adjusted for sex, age, highest attainted educational level at age 50 years, and living status (living alone, living with someone, or at nursing home) at index date.

ATC: Anatomical Therapeutic Chemical.

**Table S4. Sensitivity analysis by dementia syndrome severity at time of diagnosis – incidence rate ratio by disease category in three time-intervals**

| ICD-10 Code range and chapters Time Period | | | Mild dementia | | Moderate/severe dementia | |
| --- | --- | --- | --- | --- | --- | --- |
|  |  |  | IRR 95% CI | | IRR 95% CI | |
| A00-B99 |  |  |  |  |  |  |
| Certain infections | | 10 - >5 years prior | 0.89 | 0.78 – 1.01 | 0.93 | 0.83 – 1.04 |
|  |  | 5 - > 1 years prior | 1.03 | 0.93 – 1.14 | 1.04 | 0.95 – 1.14 |
|  |  | < 1 year prior | 1.17 | 1.02 – 1.34 | 1.60 | 1.43 – 1.79 |
| C00-D48 |  |  |  |  |  |  |
| Neoplasms | | 10 - >5 years prior | 1.02 | 0.96 – 1-09 | 0.99 | 0.93 – 1.06 |
|  |  | 5 - > 1 years prior | 0.94 | 0.88 – 1.00 | 0.92 | 0.87 – 0.98 |
|  |  | < 1 year prior | 0.87 | 0.80 – 0.94 | 0.84 | 0.77 – 0.91 |
| D50-D89 |  |  |  |  |  |  |
| Hematological/Immunological | | 10 - >5 years prior | 0.93 | 0.80 – 1.08 | 1.01 | 0.89 – 1.14 |
|  |  | 5 - > 1 years prior | 0.96 | 0.85 – 1.10 | 0.97 | 0.87 – 1.08 |
|  |  | < 1 year prior | 1.21 | 1.01 – 1.43 | 1.56 | 1.36 – 1.79 |
| E00-E90 |  |  |  |  |  |  |
| Endocrine/metabolic | | 10 - >5 years prior | 1.03 | 0.97 – 1.10 | 0.98 | 0.92 – 1.04 |
|  |  | 5 - > 1 years prior | 1.00 | 0.94 – 1.07 | 1.05 | 0.99 – 1.11 |
|  |  | < 1 year prior | 2.17 | 2.00 – 2.34 | 2.38 | 2.22 – 2.55 |
| F00-F99 |  |  |  |  |  |  |
| Mental and behavioural* | | 10 - >5 years prior | 1.06 | 0.93 – 1.22 | 1.28 | 1.14 – 1.44 |
|  |  | 5 - > 1 years prior | 1.78 | 1.59 – 2.00 | 1.78 | 1.61 – 1.98 |
|  |  | < 1 year prior | 6.27 | 5.46 – 7.20 | 6.21 | 5.49 – 7.01 |
| G00-G99 |  |  |  |  |  |  |
| Nervous system | | 10 - >5 years prior | 1.07 | 0.98 – 1.17 | 0.94 | 0.87 – 1.03 |
|  |  | 5 - > 1 years prior | 1.07 | 0.98 – 1.16 | 0.89 | 0.82 – 0.97 |
|  |  | < 1 year prior | 1.64 | 1.47 – 1.83 | 1.42 | 1.27 – 1.59 |
| H00-H59 |  |  |  |  |  |  |
| Eye and adnexa | | 10 - >5 years prior | 1.10 | 1.03 – 1.17 | 0.98 | 0.93 – 1.04 |
|  |  | 5 - > 1 years prior | 1.00 | 0.94 – 1.07 | 0.92 | 0.87 – 0.98 |
|  |  | < 1 year prior | 1.03 | 0.94 – 1.13 | 1.03 | 0.95 – 1.12 |
| H60-H95 |  |  |  |  |  |  |
| Ear and mastoid process | | 10 - >5 years prior | 1.32 | 1.23 – 1.42 | 1.03 | 0.97 – 1.11 |
|  |  | 5 - > 1 years prior | 1.19 | 1.11 – 1.28 | 1.00 | 0.93 – 1.07 |
|  |  | < 1 year prior | 1.48 | 1.34 – 1.62 | 1.10 | 1.00 – 1.21 |
| I00-I99 |  |  |  |  |  |  |
| Circulatory system | | 10 - >5 years prior | 0.98 | 0.93 – 1.03 | 0.90 | 0.85 – 0.94 |
|  |  | 5 - > 1 years prior | 0.94 | 0.89 – 0.99 | 0.93 | 0.88 – 0.97 |
|  |  | < 1 year prior | 1.74 | 1.64 – 1.84 | 1.75 | 1.65 – 1.84 |
| J00-J99 |  |  |  |  |  |  |
| Respiratory system | | 10 - >5 years prior | 0.97 | 0.89 – 1.06 | 0.95 | 0.88 – 1.02 |
|  |  | 5 - > 1 years prior | 0.87 | 0.80 – 0.94 | 0.88 | 0.82 – 0.95 |
|  |  | < 1 year prior | 1.12 | 1.01 – 1.24 | 1.23 | 1.13 – 1.34 |
| K00-K93 |  |  |  |  |  |  |
| Digestive system | | 10 - >5 years prior | 1.04 | 0.98 – 1.11 | 1.00 | 0.94 – 1.06 |
|  |  | 5 - > 1 years prior | 1.07 | 1.01 – 1.14 | 0.98 | 0.92 – 1.03 |
|  |  | < 1 year prior | 1.09 | 1.00 – 1.19 | 1.22 | 1.13 – 1.33 |
| L00-L99 |  |  |  |  |  |  |
| Skin/subcutaneous system | | 10 - >5 years prior | 1.12 | 1.00 – 1.25 | 0.99 | 0.89 – 1.11 |
|  |  | 5 - > 1 years prior | 0.99 | 0.87 – 1.11 | 0.95 | 0.85 – 1.06 |
|  |  | < 1 year prior | 1.04 | 0.87 – 1.24 | 0.96 | 0.82 – 1.14 |
| M00-M99 |  |  |  |  |  |  |
| Musculoskeletal system | | 10 - >5 years prior | 1.13 | 1.08 – 1.19 | 1.00 | 0.95 – 1.05 |
|  |  | 5 - > 1 years prior | 1.03 | 0.98 – 1.08 | 0.94 | 0.89 – 0.98 |
|  |  | < 1 year prior | 1.21 | 1.13 – 1.29 | 1.26 | 1.18 – 1.34 |
| N00-N99 |  |  |  |  |  |  |
| Genitourinary system | | 10 - >5 years prior | 1.05 | 0.98 – 1.13 | 0.98 | 0.92 – 1.05 |
|  |  | 5 - > 1 years prior | 0.98 | 0.91 – 1.04 | 0.95 | 0.90 – 1.01 |
|  |  | < 1 year prior | 1.17 | 1.06 – 1.28 | 1.56 | 1.44 – 1.68 |
| R00-R99 |  |  |  |  |  |  |
| Symptoms/signs | | 10 - >5 years prior | 1.14 | 1.08 – 1.20 | 1.07 | 1.02 – 1.12 |
|  |  | 5 - > 1 years prior | 1.29 | 1.23 – 1.36 | 1.16 | 1.11 – 1.22 |
|  |  | < 1 year prior | 3.57 | 3.38 – 3.78 | 3.07 | 2.91 – 3.23 |
| S00-T98 |  |  |  |  |  |  |
| Injuries, poisoning, external causes | | 10 - >5 years prior | 1.20 | 1.14 – 1.27 | 1.19 | 1.06 – 1.18 |
|  |  | 5 - > 1 years prior | 1.24 | 1.18 – 1.31 | 1.24 | 1.18 – 1.30 |
|  |  | < 1 year prior | 1.46 | 1.36 – 1.57 | 1.99 | 1.87 – 2.12 |
| Z00-Z80 |  |  |  |  |  |  |
| Factors influencing health status | | 10 - >5 years prior | 1.28 | 1.21 – 1.37 | 1.02 | 0.96 – 1.08 |
|  |  | 5 - > 1 years prior | 1.30 | 1.22 – 1.38 | 1.06 | 1.00 – 1.12 |
|  |  | < 1 year prior | 13.40 | 12.33 – 14.56 | 8.93 | 8.35 – 9.56 |

Adjusted incidence rate ratios (IRRs) for late onset Alzheimer's disease cases are presented in three time-intervals prior to diagnosis with 95% confidence intervals (CI) according to dementia syndrome severity at time of diagnosis (patients with mild dementia compared to their controls, and patients with moderate/severe dementia compared to their controls). IRRs are adjusted for age, sex, highest attained educational level at age 50 years, and living status at index date (living alone, living with someone, or at nursing home).

*****Excluding mild cognitive impairment and dementia diagnosis

ICD-10: International Classification of Diseases, 10^th^ revision

**Table S5. Sensitivity analysis by dementia syndrome severity at time of diagnosis – incidence rate ratio by medication category in three time-intervals**

| Overall category | | Time Period | Mild dementia | | Moderate/severe dementia | |  |
| --- | --- | --- | --- | --- | --- | --- | --- |
|  |  |  | IRR | 95% CI | IRR | 95% CI |  |
| A |  |  |  |  |  |  |  |
| Alimentary tract and metabolism | | 10 - >5 years prior | 1.13 | 1.08 – 1.19 | 1.19 | 1.07 – 1.17 |  |
|  |  | 5 - > 1 years prior | 1.03 | 0.98 – 1.08 | 1.05 | 1.00 – 1.10 |  |
|  |  | < 1 year prior | 0.99 | 0.94 – 1.04 | 1.17 | 1.12 – 1.23 |  |
| B |  |  |  |  |  |  |  |
| Blood and blood forming organs | | 10 - >5 years prior | 1.14 | 1.09 – 1.20 | 1.11 | 1.06 – 1.16 |  |
|  |  | 5 - > 1 years prior | 1.15 | 1.09 – 1.20 | 1.15 | 1.09 – 1.20 |  |
|  |  | < 1 year prior | 1.30 | 1.24 – 1.37 | 1.38 | 1.32 – 1.45 |  |
| C |  |  |  |  |  |  |  |
| Cardiovascular system | | 10 - >5 years prior | 1.31 | 1.24 – 1.39 | 1.27 | 1.20 – 1.34 |  |
|  |  | 5 - > 1 years prior | 1.24 | 1.17 – 1.31 | 1.17 | 1.10 – 1.23 |  |
|  |  | < 1 year prior | 1.21 | 1.14 – 1.28 | 1.11 | 1.05 – 1.17 |  |
| D |  |  |  |  |  |  |  |
| Dermatologicals | | 10 - >5 years prior | 1.28 | 1.22 – 1.34 | 1.15 | 1.10 – 1.20 |  |
|  |  | 5 - > 1 years prior | 1.13 | 1.08 – 1.19 | 0.95 | 0.90 – 0.99 |  |
|  |  | < 1 year prior | 0.88 | 0.83 – 0.93 | 0.83 | 0.79 – 0.88 |  |
| G |  |  |  |  |  |  |  |
| Genital urinary system and sex hormones | | 10 - >5 years prior | 1.40 | 1.34 – 1.48 | 1.19 | 1.14 – 1.25 |  |
|  |  | 5 - > 1 years prior | 1.28 | 1.22 – 1.35 | 1.05 | 1.00 – 1.10 |  |
|  |  | < 1 year prior | 1.03 | 0.97 – 1.09 | 0.87 | 0.83 – 0.93 |  |
| H |  |  |  |  |  |  |  |
| Systemic hormonal preperations, excluding sex hormones and insulin | | 10 - >5 years prior | 1.09 | 1.03 – 1.15 | 1.05 | 0.99 – 1.11 |  |
|  |  | 5 - > 1 years prior | 0.95 | 0.89 – 1.00 | 0.98 | 0.92 – 1.03 |  |
|  |  | < 1 year prior | 0.94 | 0.88 – 1.01 | 0.93 | 0.87 – 0.99 |  |
| J |  |  |  |  |  |  |  |
| Antiinfectives for systemic use | | 10 - >5 years prior | 1.41 | 1.33 – 1.49 | 1.23 | 1.17 – 1.29 |  |
|  |  | 5 - > 1 years prior | 1.15 | 1.09 – 1.21 | 1.04 | 0.99 – 1.09 |  |
|  |  | < 1 year prior | 1.09 | 1.03 – 1.14 | 1.32 | 1.26 – 1.38 |  |
| L |  |  |  |  |  |  |  |
| Antineoplastic and immunomodulating agents | | 10 - >5 years prior | 1.41 | 1.33 – 1.49 | 1.09 | 0.93 – 1.26 |  |
|  |  | 5 - > 1 years prior | 1.15 | 1.09 – 1.21 | 1.08 | 0.93 – 1.24 |  |
|  |  | < 1 year prior | 1.09 | 1.03 – 1.14 | 1.02 | 0.86 – 1.21 |  |
| M |  |  |  |  |  |  |  |
| Musculoskeletal system | | 10 - >5 years prior | 1.28 | 1.21 – 1.34 | 1.12 | 1.07 – 1.17 |  |
|  |  | 5 - > 1 years prior | 1.13 | 1.08 – 1.19 | 0.95 | 0.91 – 1.00 |  |
|  |  | < 1 year prior | 0.97 | 0.92 – 1.03 | 0.90 | 0.86 – 0.95 |  |
| N |  |  |  |  |  |  |  |
| Nervous system |  | 10 - >5 years prior | 1.35 | 1.28 – 1.42 | 1.22 | 1.16 – 1.28 |  |
|  |  | 5 - > 1 years prior | 1.32 | 1.25 – 1.39 | 1.22 | 1.16 – 1.29 |  |
|  |  | < 1 year prior | 1.27 | 1.21 – 1.33 | 1.33 | 1.27 – 1.40 |  |
| P |  |  |  |  |  |  |  |
| Antiparasitic products, insecticides, and repellents |  | 10 - >5 years prior | 1.14 | 1.06 – 1.22 | 1.08 | 1.00 – 1.15 |  |
|  |  | 5 - > 1 years prior | 1.06 | 0.98 – 1.15 | 0.89 | 0.82 – 0.96 |  |
|  |  | < 1 year prior | 0.87 | 0.76 – 1.00 | 0.82 | 0.72 – 0.93 |  |
| R |  |  |  |  |  |  |  |
| Respiratory system |  | 10 - >5 years prior | 1.21 | 1.15 – 1.27 | 1.11 | 1.06 – 1.16 |  |
|  |  | 5 - > 1 years prior | 1.07 | 1.02 – 1.12 | 0.92 | 0.88 – 0.97 |  |
|  |  | < 1 year prior | 0.79 | 0.75 – 0.84 | 0.76 | 0.72 – 0.81 |  |
| S |  |  |  |  |  |  |  |
| Sensory organs |  | 10 - >5 years prior | 1.27 | 1.21 – 1.33 | 1.16 | 1.11 – 1.21 |  |
|  |  | 5 - > 1 years prior | 1.12 | 1.07 – 1.18 | 0.92 | 0.88 – 0.97 |  |
|  |  | < 1 year prior | 0.89 | 0.84 – 0.95 | 0.80 | 0.75 – 0.84 |  |
| Adjusted incidence rate ratios (IRRs) for late onset Alzheimer's disease cases are presented in three time-intervals prior to diagnosis with 95% confidence intervals (CI) according to dementia syndrome severity at time of diagnosis (patients with mild dementia compared to their controls, and patients with moderate/severe dementia compared to their controls). IRRs are adjusted for age, sex, highest attained educational level at age 50 years, and living status at index date (living alone, living with someone, or at nursing home). | | | | | | |  |
|  |  |  |  |  |  |  |  |
|  |  |  |  |  |  |  |  |
|  |  |  |  |  |  |  |  |

**Table S6. Sensitivity analysis by sex and age – incidence rate ratio by disease category in three time-intervals**

| ICD-10 Code range and chapters | | Time Period | Sex | | | | Age | | | |
| --- | --- | --- | --- | --- | --- | --- | --- | --- | --- | --- |
|  |  |  | Men | | Women | | ≤80 years | | > 80 years | |
|  |  |  | IRR | 95% CI | IRR | 95% CI | IRR | 95% CI | 95% CI | |
| A00-B99 |  |  |  |  |  |  |  |  |  |  |
| Certain infections | | 10 - >5 years prior | 0.99 | 0.87 – 1.12 | 0.85 | 0.76 – 0.95 | 1.00 | 0.87 – 1.14 | 0.85 | 0.77 – 0.95 |
|  |  | 5 - > 1 years prior | 1.14 | 1.04 – 1.27 | 0.94 | 0.86 – 1.03 | 1.10 | 0.98 – 1.23 | 1.00 | 0.92 – 1.09 |
|  |  | < 1 year prior | 1.48 | 1.30 – 1.69 | 1.34 | 1.19 – 1.50 | 1.65 | 1.42 – 1.91 | 1.29 | 1.16 – 1.43 |
| C00-D48 |  |  |  |  |  |  |  |  |  |  |
| Neoplasms | | 10 - >5 years prior | 1.03 | 0.96 – 1.11 | 0.98 | 0.93 – 1.04 | 1.02 | 0.95 – 1.09 | 1.00 | 0.94 – 1.06 |
|  |  | 5 - > 1 years prior | 0.95 | 0.89 – 1.02 | 0.91 | 0.86 – 0.96 | 0.94 | 0.88 – 1.00 | 0.93 | 0.88 – 0.98 |
|  |  | < 1 year prior | 0.90 | 0.82 – 0.98 | 0.81 | 0.75 – 0.88 | 0.91 | 0.83 – 0.99 | 0.81 | 0.75 – 0.87 |
| D50-D89 |  |  |  |  |  |  |  |  |  |  |
| Hematological/Immunological | | 10 - >5 years prior | 0.90 | 0.76 – 1.05 | 1.02 | 0.90 – 1.14 | 1.02 | 0.86 – 1.22 | 0.95 | 0.85 – 1.06 |
|  |  | 5 - > 1 years prior | 0.97 | 0.85 – 1.11 | 0.95 | 0.86 – 1.06 | 1.01 | 0.86 – 1.18 | 0.93 | 0.85 – 1.03 |
|  |  | < 1 year prior | 1.31 | 1.10 – 1.56 | 1.46 | 1.28 – 1.68 | 1.49 | 1.22 – 1.81 | 1.36 | 1.20 – 1.54 |
| E00-E90 |  |  |  |  |  |  |  |  |  |  |
| Endocrine/metabolic | | 10 - >5 years prior | 0.98 | 0.91 – 1.06 | 1.01 | 0.95 – 1.06 | 1.08 | 1.00 – 1.16 | 0.94 | 0.89 – 1.00 |
|  |  | 5 - > 1 years prior | 1.00 | 0.93 – 1.08 | 1.03 | 0.97 – 1.08 | 1.08 | 1.00 – 1.15 | 0.99 | 0.93 – 1.04 |
|  |  | < 1 year prior | 1.99 | 1.83 – 2.17 | 2.45 | 12.30 – 2.61 | 2.42 | 2.22 – 2.63 | 2.19 | 2.05 – 2.34 |
| F00-F99 |  |  |  |  |  |  |  |  |  |  |
| Mental and behavioural* | | 10 - >5 years prior | 1.17 | 1.01 – 1.37 | 1.16 | 1.04 – 1.29 | 1.32 | 1.16 – 1.50 | 1.03 | 0.91 – 1.16 |
|  |  | 5 - > 1 years prior | 1.71 | 1.50 – 1.95 | 1.78 | 1.62 – 1.95 | 2.19 | 1.95 – 2.47 | 1.47 | 1.33 – 1.62 |
|  |  | < 1 year prior | 6.49 | 5.54 – 7.61 | 5.94 | 5.32 – 6.64 | 8.43 | 7.20 – 9.87 | 5.10 | 4.55 – 5.71 |
| G00-G99 |  |  |  |  |  |  |  |  |  |  |
| Nervous system | | 10 - >5 years prior | 0.94 | 0.85 – 1.04 | 1.01 | 0.94 – 1.10 | 1.01 | 0.92 – 1.12 | 0.96 | 0.89 – 1.04 |
|  |  | 5 - > 1 years prior | 1.06 | 0.97 – 1.16 | 0.90 | 0.83 – 0.97 | 1.11 | 1.02 – 1.22 | 0.86 | 0.80 – 0.93 |
|  |  | < 1 year prior | 1.64 | 1.46 – 1.85 | 1.40 | 1.26 – 1.56 | 1.71 | 1.52 – 1.93 | 1.34 | 1.20 – 1.49 |
| H00-H59 |  |  |  |  |  |  |  |  |  |  |
| Eye and adnexa | | 10 - >5 years prior | 1.04 | 0.96 – 1.12 | 1.03 | 0.98 – 1.08 | 1.02 | 0.94 – 1.10 | 1.04 | 0.99 – 1.10 |
|  |  | 5 - > 1 years prior | 0.98 | 0.91 – 1.05 | 0.94 | 0.89 – 0.99 | 1.00 | 0.93 – 1.07 | 0.93 | 0.88 – 0.98 |
|  |  | < 1 year prior | 1.16 | 1.04 – 1.28 | 0.97 | 0.90 – 1.05 | 1.10 | 0.99 – 1.22 | 0.99 | 0.92 – 1.07 |
| H60-H95 |  |  |  |  |  |  |  |  |  |  |
| Ear and mastoid process | | 10 - >5 years prior | 1.23 | 1.15 – 1.33 | 1.10 | 1.03 – 1.18 | 1.22 | 1.12 – 1.34 | 1.13 | 1.07 – 1.20 |
|  |  | 5 - > 1 years prior | 1.20 | 1.11 – 1.29 | 1.01 | 0.95 – 1.08 | 1.10 | 1.01 – 1.20 | 1.09 | 1.02 – 1.15 |
|  |  | < 1 year prior | 1.39 | 1.26 – 1.54 | 1.19 | 1.09 – 1.30 | 1.22 | 1.08 – 1.37 | 1.31 | 1.20 – 1.41 |
| I00-I99 |  |  |  |  |  |  |  |  |  |  |
| Circulatory system | | 10 - >5 years prior | 0.90 | 0.85 – 0.95 | 0.95 | 0.91 – 0.99 | 1.00 | 0.94 – 1.06 | 0.89 | 0.85 – 0.93 |
|  |  | 5 - > 1 years prior | 0.92 | 0.87 – 0.98 | 0.93 | 0.89 – 0.97 | 1.00 | 0.94 – 1.05 | 0.89 | 0.85 – 0.93 |
|  |  | < 1 year prior | 1.58 | 1.49 – 1.68 | 1.85 | 1.76 – 1.95 | 1.75 | 1.64 – 1.87 | 1.74 | 1.66 – 1.83 |
| J00-J99 |  |  |  |  |  |  |  |  |  |  |
| Respiratory system | | 10 - >5 years prior | 0.93 | 0.85 – 1.02 | 0.96 | 0.89 – 1.03 | 1.00 | 0.91 – 1.10 | 0.92 | 0.86 – 0.99 |
|  |  | 5 - > 1 years prior | 0.90 | 0.83 – 0 98 | 0.85 | 0.80 – 0.91 | 0.92 | 0.84 – 1.00 | 0.85 | 0.80 – 0.90 |
|  |  | < 1 year prior | 1.24 | 1.12 – 1.37 | 1.14 | 1.05 – 1.24 | 1.38 | 1.24 – 1.54 | 1.07 | 0.99 – 1.16 |
| K00-K93 | |  |  |  |  |  |  |  |  |  |
| Digestive system | | 10 - >5 years prior | 1.03 | 0.96 – 1.10 | 1.01 | 0.95 – 1.06 | 1.03 | 0.97 – 1.11 | 1.00 | 0.95 – 1.06 |
|  |  | 5 - > 1 years prior | 1.07 | 1.00 – 1.14 | 0.99 | 0.94 – 1.04 | 1.05 | 0.99 – 1.12 | 0.99 | 0.94 – 1.05 |
|  |  | < 1 year prior | 1.22 | 1.11 – 1.34 | 1.12 | 1.04 – 1.21 | 1.13 | 1.02 – 1.24 | 1.19 | 1.10 – 1.28 |
| L00-L99 |  |  |  |  |  |  |  |  |  |  |
| Skin/subcutaneous system | | 10 - >5 years prior | 1.14 | 1.00 – 1.30 | 0.99 | 0.90 – 1.10 | 1.02 | 0.91 – 1.16 | 1.06 | 0.95 – 1.17 |
|  |  | 5 - > 1 years prior | 0.99 | 0.88 – 1.12 | 0.95 | 0.86 – 1.05 | 0.93 | 0.82 – 1.06 | 0.99 | 0.90 – 1.09 |
|  |  | < 1 year prior | 0.94 | 0.78 – 1.14 | 1.02 | 0.88 – 1.19 | 1.12 | 0.92 – 1.35 | 0.92 | 0.79 – 1.08 |
| M00-M99 |  |  |  |  |  |  |  |  |  |  |
| Musculoskeletal system | | 10 - >5 years prior | 1.05 | 0.99 – 1.12 | 1.06 | 1.02 – 1.11 | 1.06 | 1.00 – 1.12 | 1.06 | 1.01 – 1.11 |
|  |  | 5 - > 1 years prior | 1.02 | 0.96 – 1.08 | 0.96 | 0.92 – 1.01 | 0.96 | 0.91 – 1.02 | 1.00 | 0.95 – 1.04 |
|  |  | < 1 year prior | 1.23 | 1.13 – 1.33 | 1.25 | 1.18 – 1.31 | 1.11 | 1.03 – 1.19 | 1.34 | 1.26 – 1.42 |
| N00-N99 |  |  |  |  |  |  |  |  |  |  |
| Genitourinary system | | 10 - >5 years prior | 1.01 | 0.94 – 1.09 | 1.01 | 0.95 – 1.07 | 1.05 | 0.97 – 1.13 | 0.99 | 0.93 – 1.05 |
|  |  | 5 - > 1 years prior | 0.94 | 0.87 – 1.01 | 0.98 | 0.92 – 1.03 | 0.92 | 0.86 – 1.00 | 0.99 | 0.93 – 1.04 |
|  |  | < 1 year prior | 1.32 | 1.20 – 1.45 | 1.41 | 1.31 – 1.52 | 1.27 | 1.15 – 1.41 | 1.43 | 1.33 – 1.53 |
| R00-R99 |  |  |  |  |  |  |  |  |  |  |
| Symptoms/signs | | 10 - >5 years prior | 1.09 | 1.03 – 1.16 | 1.10 | 1.05 – 1.15 | 1.15 | 1.08 – 1.22 | 1.06 | 1.02 – 1.12 |
|  |  | 5 - > 1 years prior | 1.21 | 1.14 – 1.28 | 1.22 | 1.16 – 1.27 | 1.29 | 1.23 – 1.37 | 1.16 | 1.11 – 1.21 |
|  |  | < 1 year prior | 3.21 | 3.02 – 3.41 | 3.33 | 3.17 – 3.50 | 4.11 | 3.86 – 4.37 | 2.84 | 2.70 – 2.98 |
| S00-T98 |  |  |  |  |  |  |  |  |  |  |
| Injuries, poisoning, external causes | | 10 - >5 years prior | 1.18 | 1.11 – 1.26 | 1.13 | 1.08 – 1.18 | 1.21 | 1.14 – 1.28 | 1.11 | 1.06 – 1.17 |
|  |  | 5 - > 1 years prior | 1.26 | 1.18 – 1.33 | 1.23 | 1.17 – 1.28 | 1.29 | 1.22 – 1.37 | 1.21 | 1.15 – 1.26 |
|  |  | < 1 year prior | 1.65 | 1.52 – 1.79 | 1.78 | 1.68 – 1.88 | 1.70 | 1.57 – 1.84 | 1.75 | 1.65 – 1.85 |
| Z00-Z80 |  |  |  |  |  |  |  |  |  |  |
| Factors influencing health status | | 10 - >5 years prior | 1.10 | 1.04 – 1.17 | 1.15 | 1.08 – 1.21 | 1.16 | 1.08 – 1.25 | 1.11 | 1.06 – 1.17 |
|  |  | 5 - > 1 years prior | 1.21 | 1.14 – 1.29 | 1.12 | 1.07 – 1.18 | 1.25 | 1.17 – 1.33 | 1.11 | 1.05 – 1.17 |
|  |  | < 1 year prior | 12.82 | 11.69 – 14.05 | 9.59 | 9.00 – 10.21 | 12.24 | 11.24 – 13.32 | 9.67 | 9.05 – 10.33 |

Adjusted incidence rate ratios (IRRs) for late onset Alzheimer's disease cases are presented in three time-intervals prior to diagnosis with 95% confidence intervals (CI) according to sex and age. IRRs are adjusted for age, sex, highest attained educational level at age 50 years, and living status at index date (living alone, living with someone, or at nursing home).

*****Excluding mild cognitive impairment and dementia diagnosis

ICD-10: International Classification of Diseases, 10^th^ revision

**Table S7. Sensitivity analysis by sex and age – incidence rate ratio by medication category in three time-intervals**

| Overall category | | Time Period | Sex | | | | Age | | | |  |
| --- | --- | --- | --- | --- | --- | --- | --- | --- | --- | --- | --- |
|  |  |  | Men | | Women | | ≤ 80 years | | > 80 years | |  |
|  |  |  | IRR | 95% CI | IRR | 95% CI | IRR | 95% CI | IRR | 95% CI |  |
| A |  |  |  |  |  |  |  |  |  |  |  |
| Alimentary tract and metabolism | | 10 - >5 years prior | 1.07 | 1.02 – 1.13 | 1.15 | 1.02 – 1.20 | 1.12 | 1.07 – 1.18 | 1.12 | 1.07 – 1.17 |  |
|  |  | 5 - > 1 years prior | 1.03 | 0.97 – 1.08 | 1.04 | 1.00 – 1.08 | 1.04 | 0.99 – 1.10 | 1.02 | 0.98 – 1.07 |  |
|  |  | < 1 year prior | 1.04 | 0.99 – 1.10 | 1.10 | 1.05 – 1.15 | 1.05 | 1.00 – 1.11 | 1.09 | 1.04 – 1.14 |  |
| B |  |  |  |  |  |  |  |  |  |  |  |
| Blood and blood forming organs | | 10 - >5 years prior | 1.10 | 1.04 – 1.16 | 1.14 | 1.09 – 1.19 | 1.16 | 1.10 – 1.23 | 1.09 | 1.05 – 1.14 |  |
|  |  | 5 - > 1 years prior | 1.13 | 1.07 – 1.20 | 1.15 | 1.10 – 1.20 | 1.19 | 1.13 – 1.26 | 1.11 | 1.06 – 1.16 |  |
|  |  | < 1 year prior | 1.31 | 1.24 – 1.39 | 1.35 | 1.30 – 1.41 | 1.40 | 1.33 – 1.48 | 1.30 | 1.24 – 1.35 |  |
| C |  |  |  |  |  |  |  |  |  |  |  |
| Cardiovascular system | | 10 - >5 years prior | 1.21 | 1.13 – 1.28 | 1.33 | 1.27 – 1.40 | 1.25 | 1.18 – 1.32 | 1.32 | 1.26 – 1.40 |  |
|  |  | 5 - > 1 years prior | 1.12 | 1.05 – 1.20 | 1.25 | 1.18 – 1.31 | 1.18 | 1.12 – 1.26 | 1.21 | 1.15 – 1.28 |  |
|  |  | < 1 year prior | 1.11 | 1.05 – 1.19 | 1.18 | 1.12 – 1.23 | 1.25 | 1.18 – 1.32 | 1.08 | 1.03 – 1.14 |  |
| D |  |  |  |  |  |  |  |  |  |  |  |
| Dermatologicals | | 10 - >5 years prior | 1.14 | 1.08 – 1.20 | 1.24 | 1.19 – 1.30 | 1.15 | 1.09 – 1.21 | 1.25 | 1.20 – 1.31 |  |
|  |  | 5 - > 1 years prior | 1.02 | 0.97 – 1.08 | 1.03 | 0.99 – 1.07 | 1.00 | 0.95 – 1.05 | 1.05 | 1.01 – 1.10 |  |
|  |  | < 1 year prior | 0.87 | 0.82 – 0.93 | 0.84 | 0.80 – 0.88 | 0.84 | 0.78 – 0.89 | 0.86 | 0.82 – 0.91 |  |
| G |  |  |  |  |  |  |  |  |  |  |  |
| Genital urinary system and sex hormones | | 10 - >5 years prior | 1.26 | 1.19 – 1.33 | 1.31 | 1.25 – 1.37 | 1.21 | 1.15 – 1.28 | 1.35 | 1.29 – 1.41 |  |
|  |  | 5 - > 1 years prior | 1.19 | 1.12 – 1.26 | 1.14 | 1.09 – 1.20 | 1.08 | 1.02 – 1.14 | 1.22 | 1.16 – 1.28 |  |
|  |  | < 1 year prior | 1.09 | 1.02 – 1.15 | 0.87 | 0.82 – 0.91 | 0.85 | 0.80 – 0.90 | 1.03 | 0.98 – 1.08 |  |
| H |  |  |  |  |  |  |  |  |  |  |  |
| Systemic hormonal preperations, excluding sex hormones and insulin | | 10 - >5 years prior | 1.06 | 0.98 – 1.13 | 1.07 | 1.02 – 1.12 | 1.05 | 0.99 – 1.12 | 1.08 | 1.03 – 1.13 |  |
|  |  | 5 - > 1 years prior | 0.95 | 0.89 – 1.02 | 0.97 | 0.92 – 1.01 | 0.95 | 0.89 – 1.02 | 0.97 | 0.92 – 1.02 |  |
|  |  | < 1 year prior | 0.89 | 0.91 – 0.97 | 0.95 | 0.90 – 1.00 | 0.95 | 0.88 – 1.03 | 0.92 | 0.87 – 0.98 |  |
| J |  |  |  |  |  |  |  |  |  |  |  |
| Antiinfectives for systemic use | | 10 - >5 years prior | 1.17 | 1.10 – 1.24 | 1.41 | 1.35 – 1.48 | 1.19 | 1.12 – 1.26 | 1.40 | 1.34 – 1.48 |  |
|  |  | 5 - > 1 years prior | 1.04 | 0.96 – 1.07 | 1.13 | 1.08 – 1.18 | 1.01 | 0.96 – 1.06 | 1.15 | 1.10 – 1.21 |  |
|  |  | < 1 year prior | 1.10 | 1.04 – 1.16 | 1.26 | 1.21 – 1.31 | 1.05 | 0.99 – 1.11 | 1.31 | 1.26 – 1.37 |  |
| L |  |  |  |  |  |  |  |  |  |  |  |
| Antineoplastic and immunomodulating agents | | 10 - >5 years prior | 1.19 | 0.97 – 1.46 | 1.10 | 0.97 – 1.27 | 1.20 | 1.01 – 1.42 | 1.09 | 0.94 – 1.26 |  |
|  |  | 5 - > 1 years prior | 1.19 | 0.99 – 1.42 | 1.03 | 0.90 – 1.17 | 1.09 | 0.93 – 1.29 | 1.06 | 0.92 – 1.21 |  |
|  |  | < 1 year prior | 1.12 | 0.91 – 1.39 | 0.95 | 0.81 – 1.11 | 1.03 | 0.84 – 1.25 | 0.99 | 0.84 – 1.16 |  |
| M |  |  |  |  |  |  |  |  |  |  |  |
| Musculoskeletal system | | 10 - >5 years prior | 1.13 | 1.07 – 1.19 | 1.22 | 1.17 – 1.28 | 1.15 | 1.10 – 1.22 | 1.21 | 1.16 – 1.27 |  |
|  |  | 5 - > 1 years prior | 1.00 | 0.95 – 1.06 | 1.06 | 1.01 – 1.10 | 1.04 | 0.99 – 1.09 | 1.03 | 0.99 – 1.08 |  |
|  |  | < 1 year prior | 0.87 | 0.82 – 0.94 | 0.97 | 0.92 – 1.01 | 0.90 | 0.85 – 0.96 | 0.96 | 0.91 – 1.01 |  |
| N |  |  |  |  |  |  |  |  |  |  |  |
| Nervous system | | 10 - >5 years prior | 1.19 | 1.13 – 1.26 | 1.33 | 1.27 – 1.40 | 1.24 | 1.18 – 1.31 | 1.30 | 1.24 – 1.37 |  |
|  |  | 5 - > 1 years prior | 1.18 | 1.11 – 1.25 | 1.34 | 1.27 – 1.40 | 1.23 | 1.16 – 1.30 | 1.30 | 1.24 – 1.37 |  |
|  |  | < 1 year prior | 1.24 | 1.17 – 1.31 | 1.34 | 1.28 – 1.40 | 1.35 | 1.29 – 1.43 | 1.25 | 1.20 – 1.31 |  |
| P |  |  |  |  |  |  |  |  |  |  |  |
| Antiparasitic products, insecticides, and repellents | | 10 - >5 years prior | 1.13 | 1.04 – 1.24 | 1.09 | 1.03 – 1.16 | 1.05 | 0.97 – 1.13 | 1.16 | 1.08 – 1.23 |  |
|  |  | 5 - > 1 years prior | 0.98 | 0.88 – 1.08 | 0.96 | 0.90 – 1.03 | 0.99 | 0.90 – 1.08 | 0.95 | 0.88 – 1.02 |  |
|  |  | < 1 year prior | 0.93 | 0.79 – 1.09 | 0.81 | 0.72 – 0.90 | 0.88 | 0.76 – 1.03 | 0.82 | 0.73 – 0.92 |  |
| R |  |  |  |  |  |  |  |  |  |  |  |
| Respiratory system | | 10 - >5 years prior | 1.09 | 1.03 – 1.15 | 1.18 | 1.14 – 1.24 | 1.11 | 1.06 – 1.17 | 1.17 | 1.12 – 1.22 |  |
|  |  | 5 - > 1 years prior | 1.00 | 0.95 – 1.06 | 0.98 | 0.94 – 1.02 | 0.98 | 0.93 – 1.04 | 0.99 | 0.95 – 1.04 |  |
|  |  | < 1 year prior | 0.83 | 0.78 – 0.89 | 0.75 | 0.71 – 0.78 | 0.77 | 0.73 – 0.82 | 0.78 | 0.74 – 0.82 |  |
| S |  |  |  |  |  |  |  |  |  |  |  |
| Sensory organs | | 10 - >5 years prior | 1.17 | 1.11 – 1.24 | 1.23 | 1.18 – 1.28 | 1.12 | 1.06 – 1.18 | 1.28 | 1.22 – 1.33 |  |
|  |  | 5 - > 1 years prior | 0.98 | 0.93 – 1.04 | 1.03 | 0.99 – 1.07 | 0.97 | 0.92 – 1.03 | 1.04 | 0.99 – 1.08 |  |
|  |  | < 1 year prior | 0.90 | 0.84 – 0.96 | 0.81 | 0.77 – 0.85 | 0.83 | 0.78 – 0.89 | 0.84 | 0.80 – 0.89 |  |
| Adjusted incidence rate ratios (IRRs) for late onset Alzheimer's disease cases are presented in three time-intervals prior to diagnosis with 95% confidence intervals (CI) according to sex and age. IRRs are adjusted for age, sex, highest attained educational level at age 50 years, and living status at index date (living alone, living with someone, or at nursing home). | | | | | | | | | | |  |
|  |  |  |  |  |  |  |  |  |  |  |  |
|  |  |  |  |  |  |  |  |  |  |  |  |
|  |  |  |  |  |  |  |  |  |  |  |  |

**Table S8. Sensitivity analysis censoring contacts 6 months before index date – Incidence rate ratios by disease category in the time-interval <1- 6 months prior to diagnosis of late-onset Alzheimer’s disease**

| ICD-10 range and chapters | IRR | 95% CI |
| --- | --- | --- |
| A00-B99 Certain infections | 1.30 | 1.15 – 1.47 |
| C00-D48 Neoplasms | 0.79 | 0.74 – 0.86 |
| D50-D89 Hematological/Immunological | 1.18 | 1.02 – 1.38 |
| E00-E90 Endocrine/metabolic | 1.59 | 1.48 – 1.71 |
| F00-F99 Mental and behavioural* | 4.91 | 4.32 – 5.57 |
| G00-G99 Nervous system | 1.12 | 1.00 – 1.26 |
| H00-H59 Eye and adnexa | 0.88 | 0.82 – 0.96 |
| H60-H95 Ear and mastoid process | 1.08 | 0.99 – 1.18 |
| I00-I99 Circulatory system | 1.14 | 1.08 – 1.20 |
| J00-J99 Respiratory system | 1.06 | 0.97 – 1.16 |
| K00-K93 Digestive system | 1.05 | 0.97 – 1.14 |
| L00-L99 Skin/subcutaneous system | 0.93 | 0.79 – 1.09 |
| M00-M99 Musculoskeletal system | 1.06 | 0.99 – 1.12 |
| N00-N99 Genitourinary system | 1.12 | 1.12 – 1.31 |
| R00-R99 Symptoms/signs | 1.85 | 1.76 – 1.94 |
| S00-T98 Injuries, poisoning, external causes | 1.55 | 1.52 – 1.72 |
| Z00-Z80 Factors influencing health status | 1.75 | 1.69 – 1.81 |
|  |  |  |
|  |  |  |
|  |  |  |
|  |  |  |

*****Excluding mild cognitive impairment and dementia diagnosis
ICD-10: International Classification of diseases, 10^th^ revision

**Table S9.** Sensitivity analysis censoring contacts 6 months before index date – Incidence rate ratios by medication category in the time-interval <1- 6 months prior to diagnosis of late-onset Alzheimer’s disease

| Overall category | IRR | 95% CI |
| --- | --- | --- |
| A Alimentary tract and metabolism | 0.95 | 0.92 – 0.99 |
| B Blood and blood forming organs | 1.10 | 1.07 – 1.14 |
| C Cardiovascular system | 1.01 | 0.97 – 1.04 |
| D Dermatologicals | 0.85 | 0.81 – 0.89 |
| G Genital urinary system and sex hormones | 0.94 | 0.90 – 0.98 |
| H Systemic hormonal reparations, excluding sex hormones and insulin | 0.95 | 0.90 – 1.00 |
| J Antiinfectives for systemic use | 1.02 | 0.98 – 1.06 |
| L Antineoplastic and immunomodulating agents | 1.00 | 0.86 – 1.16 |
| M Musculoskeletal system | 0.93 | 0.89 – 0.97 |
| N Nervous system | 1.10 | 1.07 – 1.14 |
| PAntiparasitic products, insecticides, and repellents | 0.88 | 0.79 – 0.97 |
| R Respiratory system | 0.77 | 0.73 – 0.80 |
| S Sensory organs | 0.84 | 0.80 – 0.88 |

Incidence rate ratios (IRRs) for late onset Alzheimer's disease cases are presented in the time-period <1 year to 6 months prior to diagnosis with 95% confidence intervals (CI). The adjusted IRRs are adjusted for age, sex, highest attained educational level at age 50 years, and living status at index date (living alone, living with someone, or at nursing home).

**Table S10. Main analysis – unadjusted incidence rate ratios by overall disease categories in three time-intervals**

| ICD-10 range and chapters |  | Time period | Unadjusted IRR | 95% CI |
| --- | --- | --- | --- | --- |
| A00-B99 |  |  |  |  |
| Certain infections | | 10 - >5 years prior | 0.94 | 0.87 – 1.02 |
|  |  | 5 - > 1 years prior | 1.08 | 1.01 – 1.16 |
|  |  | < 1 year prior | 1.48 | 1.36 – 1.61 |
| C00-D48 |  |  |  |  |
| Neoplasms | | 10 - >5 years prior | 1.00 | 0.96 – 1.05 |
|  |  | 5 - > 1 years prior | 0.93 | 0.89 – 0.97 |
|  |  | < 1 year prior | 0.86 | 0.81 – 0.91 |
| D50-D89 |  |  |  |  |
| Hematological/Immunological | | 10 - >5 years prior | 1.01 | 0.92 – 1.11 |
|  |  | 5 - > 1 years prior | 1.00 | 0.92 – 1.08 |
|  |  | < 1 year prior | 1.46 | 1.31 – 1.62 |
| E00-E90 |  |  |  |  |
| Endocrine/metabolic | | 10 - >5 years prior | 1.02 | 0.98 – 1.07 |
|  |  | 5 - > 1 years prior | 1.06 | 1.01 – 1.10 |
|  |  | < 1 year prior | 2.34 | 2.22 – 2.46 |
| F00-F99 |  |  |  |  |
| Mental and behavioural* | | 10 - >5 years prior | 1.26 | 1.16 – 1.37 |
|  |  | 5 - > 1 years prior | 1.92 | 1.78 – 2.07 |
|  |  | < 1 year prior | 6.53 | 5.96 – 7.14 |
| G00-G99 |  |  |  |  |
| Nervous system | | 10 - >5 years prior | 1.01 | 0.95 – 1.08 |
|  |  | 5 - > 1 years prior | 0.99 | 0.93 – 1.05 |
|  |  | < 1 year prior | 1.54 | 1.42 – 1.66 |
| H00-H59 |  |  |  |  |
| Eye and adnexa | | 10 - >5 years prior | 1.02 | 0.96 – 1.08 |
|  |  | 5 - > 1 years prior | 0.95 | 0.91 – 0.99 |
|  |  | < 1 year prior | 1.02 | 0.96 – 1.08 |
| H60-H95 |  |  |  |  |
| Ear and mastoid process | | 10 - >5 years prior | 1.16 | 1.10 – 1.22 |
|  |  | 5 - > 1 years prior | 1.08 | 1.03 – 1.14 |
|  |  | < 1 year prior | 1.25 | 1.17 – 1.34 |
| S00-T98 |  |  |  |  |
| Injuries, poisoning, external causes | | 10 - >5 years prior | 1.17 | 1.13 – 1.21 |
|  |  | 5 - > 1 years prior | 1.27 | 1.23 – 1.32 |
|  |  | < 1 year prior | 1.80 | 1.72 – 1.89 |
| H60-H95 |  |  |  |  |
| Ear and mastoid process |  | 10 - >5 years prior | 1.16 | 1.10 – 1.22 |
|  |  | 5 - > 1 years prior | 1.08 | 1.03 – 1.14 |
|  |  | < 1 year prior | 1.25 | 1.17 – 1.34 |
| I00-I99 |  |  |  |  |
| Circulatory system |  | 10 - >5 years prior | 0.94 | 0.91 – 0.98 |
|  |  | 5 - > 1 years prior | 0.95 | 0.92 – 0.98 |
|  |  | < 1 year prior | 1.76 | 1.70 – 1.83 |
| J00-J99 |  |  |  |  |
| Respiratory system |  | 10 - >5 years prior | 0.97 | 0.92 – 1.03 |
|  |  | 5 - > 1 years prior | 0.91 | 0.96 – 0.95 |
|  |  | < 1 year prior | 1.23 | 1.15 – 1.31 |
| K00-K93 |  |  |  |  |
| Digestive system |  | 10 - >5 years prior | 1.02 | 0.98 – 1.06 |
|  |  | 5 - > 1 years prior | 1.03 | 0.99 – 1.07 |
|  |  | < 1 year prior | 1.17 | 1.11 – 1.25 |
| L00-L99 |  |  |  |  |
| Skin/subcutaneous system |  | 10 - >5 years prior | 1.06 | 0.98 – 1.15 |
|  |  | 5 - > 1 years prior | 0.99 | 0.91 – 1.06 |
|  |  | < 1 year prior | 1.02 | 0.91 – 1.15 |
| M00-M99 |  |  |  |  |
| Musculoskeletal system |  | 10 - >5 years prior | 1.06 | 1.03 – 1.10 |
|  |  | 5 - > 1 years prior | 0.98 | 0.95 – 1.02 |
|  |  | < 1 year prior | 1.25 | 1.19 – 1.31 |
| N00-N99 |  |  |  |  |
| Genitourinary system |  | 10 - >5 years prior | 1.03 | 0.99 – 1.08 |
|  |  | 5 - > 1 years prior | 1.00 | 0.95 – 1.04 |
|  |  | < 1 year prior | 1.43 | 1.35 – 1.51 |
| R00-R99 |  |  |  |  |
| Symptoms/signs |  | 10 - >5 years prior | 1.12 | 1.08 – 1.16 |
|  |  | 5 - > 1 years prior | 1.25 | 1.21 – 1.29 |
|  |  | < 1 year prior | 3.34 | 3.21 – 3.47 |
| Z00-Z80 |  |  |  |  |
| Factors influencing health status |  | 10 - >5 years prior | 1.13 | 1.09 – 1.18 |
|  |  | 5 - > 1 years prior | 1.17 | 1.12 – 1.22 |
|  |  | < 1 year prior | 10.48 | 9.95 – 11.03 |

Unadjusted incidence rate ratios (IRRs) for late onset Alzheimer’s disease cases are presented in three time-intervals prior to diagnosis with 95% confidence intervals (CI).

ICD-10: International Classification of diseases, 10^th^ revision
* Excluding mild cognitive impairment and dementia diagnosis

**Table S11.** Main analysis – unadjusted incidence rate ratios by overall medication categories in three time-intervals

| Overall category |  | Time period | Unadjusted IRR | 95% CI |
| --- | --- | --- | --- | --- |
| A |  |  |  |  |
| Alimentary tract and metabolism | | 10 - >5 years prior | 1.11 | 1.06 – 1.16 |
|  |  | 5 - > 1 years prior | 1.00 | 0.95 – 1.05 |
|  |  | < 1 year prior | 0.95 | 0.91 – 1.00 |
| B |  |  |  |  |
| Blood and blood forming organs | | 10 - >5 years prior | 1.12 | 1.07 – 1.18 |
|  |  | 5 - > 1 years prior | 1.12 | 1.07 – 1.18 |
|  |  | < 1 year prior | 1.27 | 1.21 – 1.34 |
| C |  |  |  |  |
| Cardiovascular system | | 10 - >5 years prior | 1.29 | 1.22 – 1.36 |
|  |  | 5 - > 1 years prior | 1.21 | 1.15 – 1.29 |
|  |  | < 1 year prior | 1.18 | 1.12 – 1.25 |
| D |  |  |  |  |
| Dermatologicals | | 10 - >5 years prior | 1.28 | 1.22 – 1.34 |
|  |  | 5 - > 1 years prior | 1.13 | 1.08 – 1.19 |
|  |  | < 1 year prior | 0.87 | 0.82 – 0.92 |
| G |  |  |  |  |
| Genital urinary system and sex hormones | | 10 - >5 years prior | 1.42 | 1.35 – 1.49 |
|  |  | 5 - > 1 years prior | 1.30 | 1.23 – 1.37 |
|  |  | < 1 year prior | 1.04 | 0.98 – 1.10 |
| H |  |  |  |  |
| Systemic hormonal preperations, excluding sex hormones and insulin | | 10 - >5 years prior | 1.08 | 1.02 – 1.14 |
|  |  | 5 - > 1 years prior | 0.94 | 0.88 – 0.99 |
|  |  | < 1 year prior | 0.93 | 0.86 – 0.99 |
| J |  |  |  |  |
| Antiinfectives for systemic use | | 10 - >5 years prior | 1.41 | 1.34 – 1.49 |
|  |  | 5 - > 1 years prior | 1.14 | 1.08 – 1.20 |
|  |  | < 1 year prior | 1.07 | 1.01 – 1.12 |
| L | |  |  |  |
| Antineoplastic and immunomodulating agents | | 10 - >5 years prior | 1.41 | 1.34 – 1.49 |
|  | | 5 - > 1 years prior | 1.14 | 1.08 – 1.20 |
|  | | < 1 year prior | 1.07 | 1.01 – 1.12 |
| M | |  |  |  |
| Musculoskeletal system | | 10 - >5 years prior | 1.27 | 1.21 – 1.33 |
|  | | 5 - > 1 years prior | 1.13 | 1.07 – 1.18 |
|  | | < 1 year prior | 0.97 | 0.91 – 1.02 |
| N | |  |  |  |
| Nervous system | | 10 - >5 years prior | 1.32 | 1.25 – 1.38 |
|  | | 5 - > 1 years prior | 1.29 | 1.23 – 1.36 |
|  | | < 1 year prior | 1.23 | 1.17 – 1.29 |
| P | |  |  |  |
| Antiparasitic products, insecticides, and repellents | | 10 - >5 years prior | 1.15 | 1.07 – 1.23 |
|  | | 5 - > 1 years prior | 1.06 | 0.98 – 1.15 |
|  | | < 1 year prior | 0.86 | 0.75 – 0.98 |
| R | |  |  |  |
| Respiratory system | | 10 - >5 years prior | 1.20 | 1.14 – 1.26 |
|  | | 5 - > 1 years prior | 1.06 | 1.01 – 1.11 |
|  | | < 1 year prior | 0.78 | 0.74 – 0.83 |
| S | |  |  |  |
| Sensory organs | | 10 - >5 years prior | 1.28 | 1.22 – 1.34 |
|  | | 5 - > 1 years prior | 1.13 | 1.07 – 1.18 |
|  | | < 1 year prior | 0.89 | 0.84 – 0.95 |

Unadjusted incidence rate ratios (IRRs) for late onset Alzheimer’s disease cases are presented in three time-intervals prior to diagnosis with 95% confidence intervals (CI).
